# Supplementary material for: Bias free multiobjective active learning for materials design and discovery
Source: Nat Commun. 2021 Apr 19;12:2312. doi: 10.1038/s41467-021-22437-0 (PMC8055971; doi:10.1038/s41467-021-22437-0)
Supplement: Supplementary file 1 — Supplementary Information [file 41467_2021_22437_MOESM1_ESM.pdf]

Supplementary Information for:
Bias free multiobjective active learning for materials design and discovery

Kevin Maik Jablonka[1], Giriprasad Melpatti Jothiappan[2], Shefang Wang[2], Berend Smit[1]✉, Brian Yoo[2]✉

[1] Laboratory of Molecular Simulation (LSMO), Institut des Sciences et Ingenierie Chimiques (ISIC), École Polytechnique Fédérale de Lausanne (EPFL), Sion, VS, Switzerland.
[2] BASF Corporation, 540 White Plains Road, Tarrytown, New York, 10591, USA.
✉ berend.smit@epfl.ch, ✉ brian.yoo@basf.com

Contents

1 Notes on active learning and Pareto dominance 2

2 Design space 9

3 Influence of composition and sequence 11

4 Coarse grained model 12

5 Molecular Simulations 14

6 Featurization 19

7 GPR surrogate model 21

8 Pareto optimal structures in feature and property space 28

9 Hyperparameter tuning for the PAL algorithm 30

10 ε-PAL Implementation 33

11 Inverse design 42

Supplementary Note 1 Notes on active learning and Pareto dominance

Problem setting considered in this work

In this work, we are interested in *classifying* with confidence a set of observations in objective space as (approximate)-Pareto optimal points or as non-dominating points, with particular focus on sampling from regions of design space near the Pareto optimal points.

Order theory

A partial order is a binary relation[1] $\succeq$ on a set Ω that satisfies

1. reflexivity : $x \succeq x$
2. antisymmetry: $x^1 \succeq x^2$ and $x^2 \succeq x^1$ imply $x = y$
3. transitivity: $x^1 \succeq x^2$ and $x^2 \succeq x^3$ imply $x^1 \succeq x^3$

for all $x^1, x^2, x^3 \in \Omega$.

The Pareto dominance relationship defines a partial order that is additionally scale and translation invariant as follows:

1. translational invariance: $\forall x \in \mathbb{R}^m : x^1 \preceq x^2 \Rightarrow x^1 + x \preceq x^2 + x$
2. scale invariance: $\forall \alpha \in \mathbb{R}^+ : x^1 \preceq x^2 \Rightarrow \alpha x^1 \preceq \alpha x^2$

In contrast, a total order is a partial order that fulfills the comparability axiom:

$$\forall x^1, x^2 \in \Omega : x^1 \preceq x^2 \vee x^2 \preceq x^1$$

A set with a total order defines a chain. To induce a total order one hence needs to introduce a bias.

Clearly, we want to remove any form of bias in (computational) materials discovery. In the initial design and discovery stage we are interested in identifying a set of possible candidates to enter the next design stage. That is, we first want to identify the partially ordered set (poset) of optimal candidates, ideally with confidence, without weighting our different objectives. In principle, this can also provide additional insights into whether certain objectives need to be weighted differently. It is important to realize that for

[1] A binary relation, R between sets X and Y, XRY, is a subset of the Cartesian product $X \times Y$

the extreme case when the number of iterations equals the number of design points, we will also be able to identify the unbiased partial order using any approach. However, in typical applications, enumerating through all iterations is unfeasible, and thus we want to use an active learning approach to keep the number of iterations as minimal as possible.

In the following sections, we illustrate how the biases in popular acquisition functions such as expected improvement can affect the resulting optimization outcome. For more detail, we refer the reader to Zitzler et al.[1], Wagner et al.[2], del Rosario et al.[3], and Moffaert and Nowé[4] (in the context of reinforcement learning).

Improvement measures

In the single-objective case it is clear how to measure improvement; it is directly given by the ranking of scalar objective values. In the multi-dimensional case, this improvement measure is no longer well-defined. Commonly used improvement measures introduce a total order in the search space and hence bias the search.

Expected Improvement

There has been an effort to generalize expected improvement (EI) measures, that are probabilistically optimal under some assumptions (see below) for multiobjective optimization. In general, expected improvement measures take the form of an integral over the product of the improvement and the probability of improvement over the non-dominated area A, which is represented by factorized normal distributions.

$$\mathrm{EI} = \int_{\mathbf{y} \in A} \underbrace{I(\mathbf{y}, \mathbf{P})}_{\text{improvement}} \underbrace{\prod_{i=1}^{m} \frac{1}{\hat{s}_i(\mathbf{x})} \phi\left(\frac{y_i(\mathbf{x}) - \hat{y}_i(\mathbf{x})}{\hat{s}_i(\mathbf{x})}\right)}_{\text{probability of improvement}} \, \mathrm{d}y_i(\mathbf{x}) \tag{1}$$

Biases with improvement measures

Only defined on a subset of the Pareto set

The simplest approach to deal with a multiobjective problem is linear scalarization, i.e., mapping the multiobjective problem into a single-objective

optimization problem. Here, we develop a weighted sum of our objectives and use this as our overall objective function:

$$L = \sum_{i}^{m} w_i y_i. \tag{2}$$

This defines a convex function. Therefore, this approach will fail for cases when parts of a Pareto front are non-convex. One can imagine performing multiple searches with different weighting functions, but this can be burdensome and it is not clear how to define such weighting functions.[5]

Dimensional imhomgenity

The first (trivial) bias is dimensional imhomegenity. This can be nicely exemplified by choosing the improvement measure as the Euclidean distance from a Pareto optimal point to the closet point in the non-dominating set as proposed by Keane[6] and also implemented by Janet et al.[7]:

$$I(\mathbf{y}, \mathbf{P}) = \min_{j=1}^{k} \sqrt{\sum_{i=1}^{m} \left(y_i(\mathbf{x}) - y_i^j\right)^2}, \tag{3}$$

where k iterates over the set of non-dominating points with the current Pareto front $\mathbf{P}$. Here, we can see that the summation can include distance metrics between observables in different dimensions. The Euclidean norm shares sensitivity to various scaling of the different objectives for all $L_{p>0}$ metrics (i.e., metrics based on a norm $\sum_i \|x_i\|^p$). This can be problematic, as we can imagine that one objective (e.g., $i = 1$) might be on the order 10^{-3}, whereas another (e.g., $i = 2$) might be on the order of 10^6. Without re-scaling, the second objective would be given a much higher weight. Hence, unless we have *a priori* knowledge of the range of values for different objectives, such re-scaling can be nontrivial. Wagner et al.[2] have shown that such a metric does not preserve the Pareto dominance relation.

In Supplementary Figure 1, we illustrate additional examples of how EI can be sensitive to such re-scaling methods.

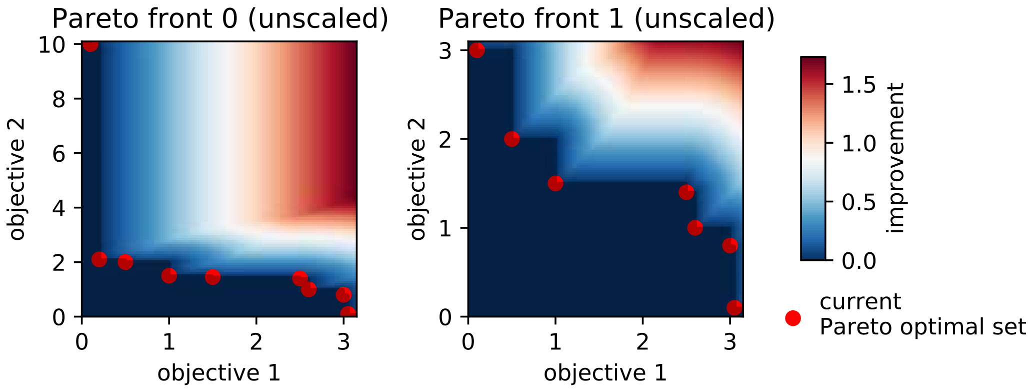

(a) Unscaled objective spaces.

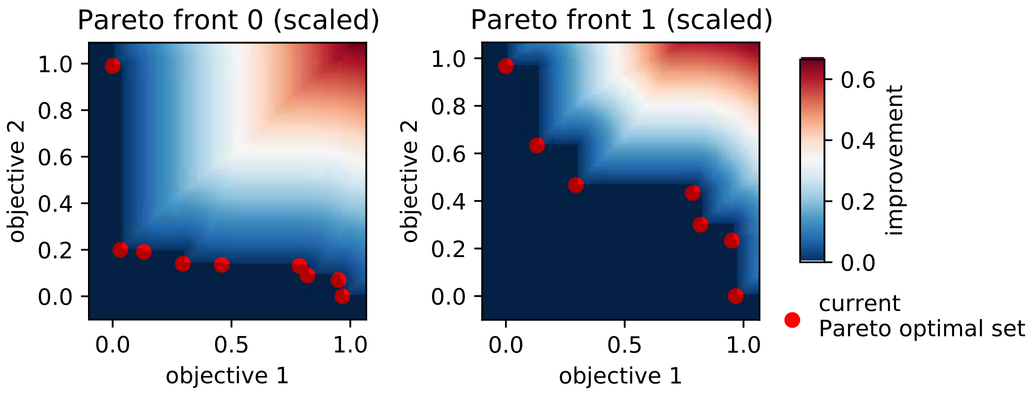

(b) Scaled objective spaces.

Supplementary Figure 1 | Improvement landscape for two fictitious Pareto frontiers using eq. 3. The color coding indicates the value of the improvement function in each grid point. In a Bayesian optimization scheme, we would choose the sample with the highest value of the improvement function, assuming that the variance is equal for all points. In a material discovery setting, however, there will not be a material on each grid point. A material with a certain combination of objectives might not exist, in which case there would be regions of space in the figure where the improvement is unknown. Note that this improvement function landscapes include "rifts" that distort the Pareto dominance relation. Also note that the improvement landscape changes when we change the scales. This is evident by comparing **a** with **b**. For example, we see for Pareto front 0 that in the unscaled space improvements in directions of objective 2 get a higher weight, wherefore the aspect ratio of the red region changes upon scaling of the objectives.

Choice of reference point

The hypervolume indicator is known as the only quantitative indicator that is strictly increasing with respect to Pareto dominance.[1] However, it can be sensitive to the choice of a reference point.

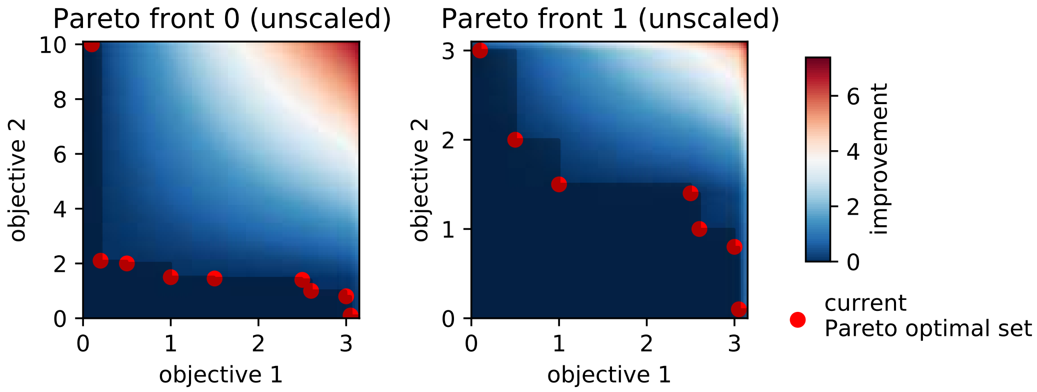

(a) Reference point [-10,-10].

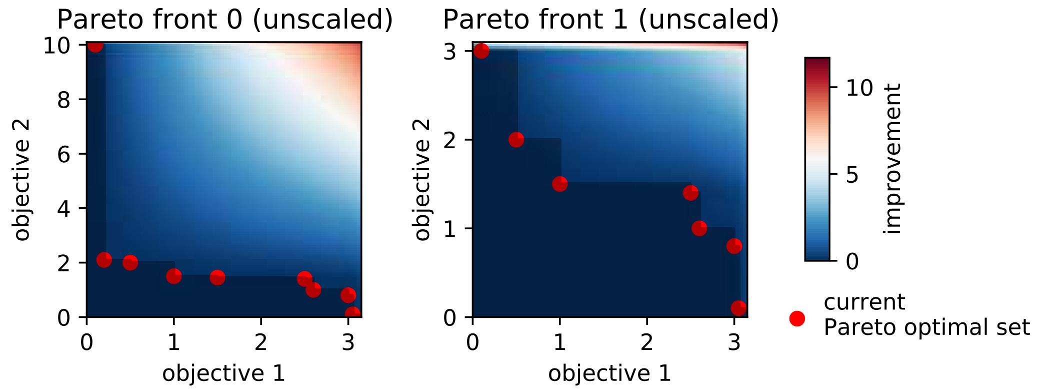

(b) Reference point [-60,-3].

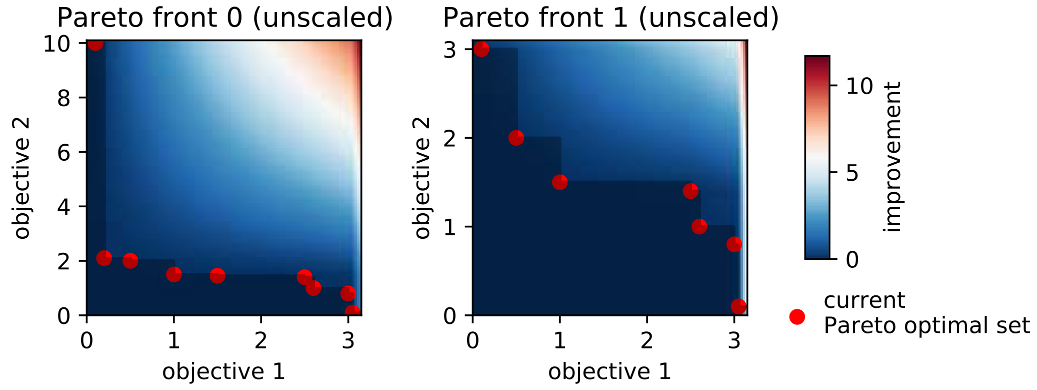

(c) Reference point [-3,-60].

Supplementary Figure 2 | Improvement function landscape based on the improvement in hypervolume, computed with different reference points for the integration of the area below the Pareto frontier. The color coding indicates the value of the improvement function in each grid point. In a Bayesian optimization scheme we would choose the sample with the highest value of the improvement function, assuming that the variance is equal for all points. In a materials discovery setting, however, there will not be a material on each grid point. A material with a certain combination of objectives might not exist, wherefore where would be blank spots in the figure.

Moreover, by construction, the hypervolume indicator gives higher weight to the convex part of the Pareto front.[1] As shown in Supplementary Figure 2, we show how improvement measures are greatest (red regions) in the top right portions of the figure panels. Regions within this space that solely improve one objective can contribute to the hypervolume indicator quite drastically depending on the reference point.[8]

Optimization vs. active learning

Expected improvement measures are probabilistically optimal under the assumption that the current sample is in the evaluated set and that the current evaluation is the final evaluation.[9] In our problem setting, however, we do not assume that the current evaluation is the final evaluation and that this evaluation will be part of the output set.[9;10] For our active learning approach, we are interested in expediting the classification of points in objective space as (approximate) Pareto-dominating points and non-dominating points. Moreover, we aim to perform this classification with tunable certainty, i.e., we want to be sure that the points we discard are with high certainty worse than the those we classify as Pareto optimal (or those that are still unclassified).

In contrast, popular optimization techniques such as Bayesian optimization and efficient global optimization (EGO) aim to find numerical solutions to a set of objective functions or a single, overall objective function obtained through scalarization. An illustration of a typical problem under Bayesian optimization with expected improvement is shown in Supplementary Figure 3. In this example, sampling the point with the highest expected improvement is likely not the optimal decision in the long run. However, this point is considered the most (locally) optimal due to the overly greedy (i.e., exploitative) nature of EI. For this reason, other metrics such as "lookahead EI" have been suggested.[11]

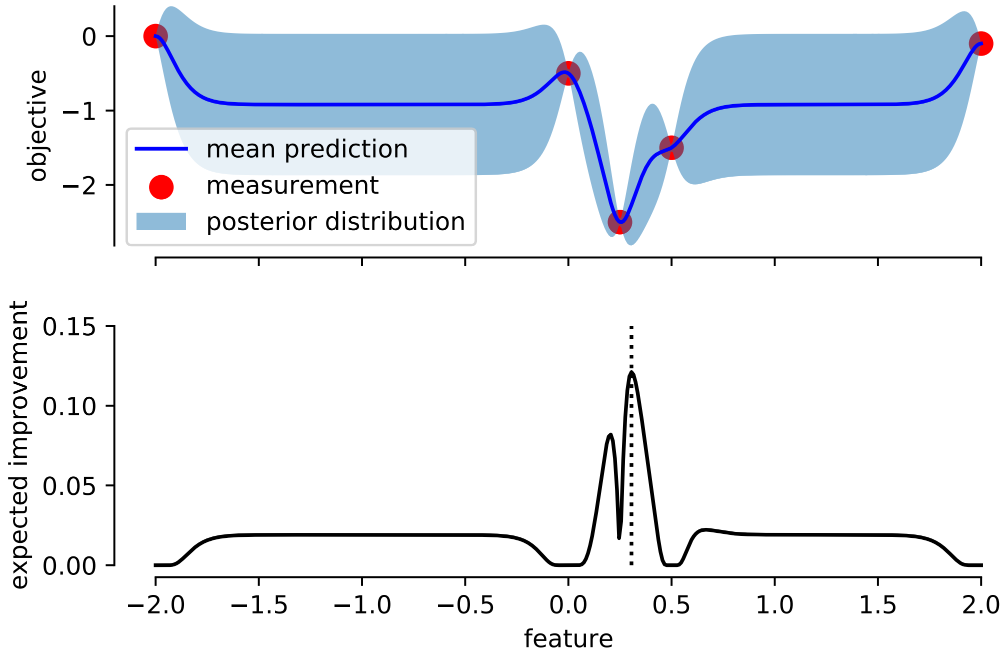

Supplementary Figure 3 | Expected improvement and predictive (posterior) distribution for a Gaussian process regression (GPR) model (Matérn-5/2 with automatic relevance determination (ARD)) on noisy samples from the Branin function.[5] Dotted line indicates the feature value that we would sample next based on the maximum value EI. In this particular case, it seems more intuitive though to take a sample in the undersampled regions with high uncertainty. Figure based on illustrations in Wu and Frazier.[11]

It is important to realize that in the limit of many samples, all optimization and active learning techniques will allow us to construct a good Pareto front; however different techniques will take different paths toward reaching this goal. Active learning techniques are more concerned with the overall information gain needed to improve the classification of optimal vs non-optimal points, whereas the aforementioned optimization approaches try to balance information gain with exploitation via an acquisition function. In the case of ϵ-Pareto active learning (PAL) the "exploitation" occurs implicitly in the "discarding" classification step (cf. Figure 4c).

Supplementary Note 2 Design space

For our design space, we considered 4 monomer types and chain lengths between 16 and 48. Furthermore, we need to consider that the reverse sequence equals the forward sequence.

The total number of polymers in our design space is then given by

$$n = \frac{1}{2} \sum_{\substack{i=16 \\ i+=1}}^{48} i^4 = 5.361\,189\,0 \times 10^7. \tag{4}$$

This results in more than 53 million possible sequences.

Enumeration is impossible for so many polymers. For example, assuming an average memory requirement of 62 kB per monomer sequence the memory footprint would correspond to 3.3 TB. This huge number of polymers also justifies our decision to use design of experiments (DoE) as an initial sampling scheme of the design space. Here, we considered increments of two, which results in a smaller design space of

$$n = \frac{1}{2} \sum_{\substack{i=16 \\ i+=2}}^{48} i^4 = 1.406\,675\,2 \times 10^7. \tag{5}$$

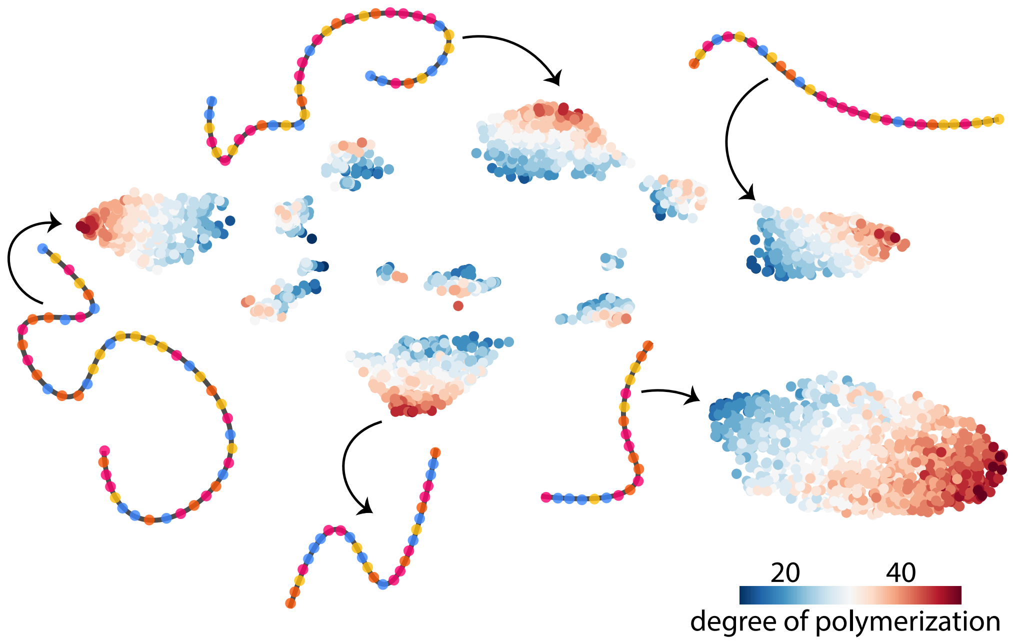

Supplementary Figure 4 | Two-dimensional projection of our polymer design space. We used the uniform manifold approximation and projection (UMAP) technique to project our design space, which we sampled using DoE, onto two dimensions. Points are colored according to the degree of polymerization. Cartoons illustrate the composition of some copolymers.

Supplementary Note 3 Influence of composition and sequence

Supplementary Figure 5 shows histograms of the standard deviation of each of the polymer descriptors at a fixed polymer composition and varying sequence. From the distributions we see that the sequence is secondary compared to the composition of the polymer as the majority of points fall within 1 standard deviation of the mean.

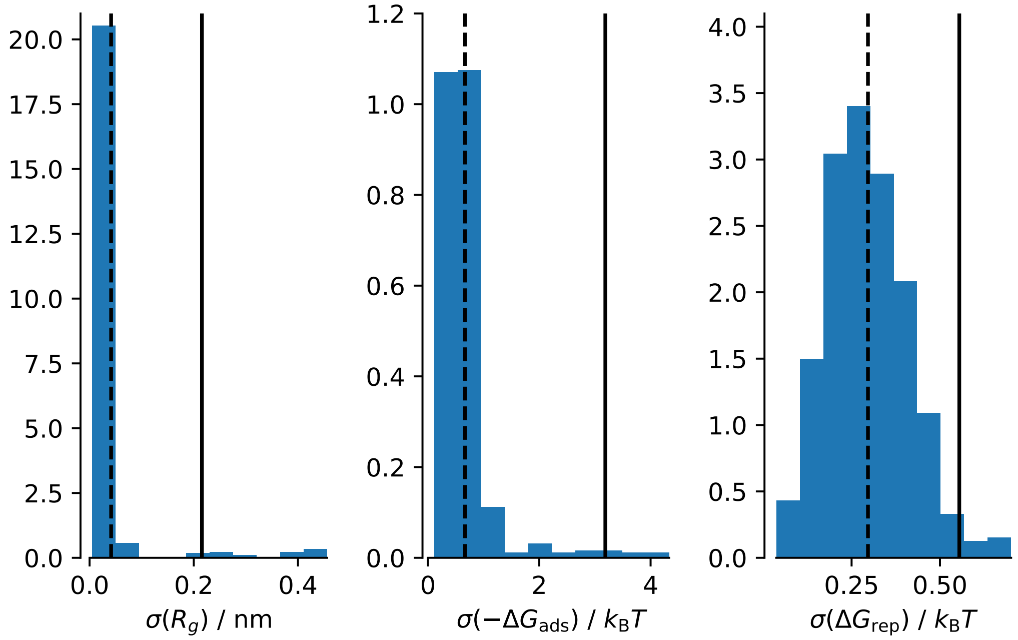

Supplementary Figure 5 | Histograms show the standard deviation of the descriptors for fixed polymer composition and varying sequence. The black solid line shows the standard deviation between the means of the descriptors for fixed composition. The dashed vertical line gives the mean standard deviation for fixed composition (means of the histograms).

Supplementary Note 4 Coarse grained model

In dissipative particle dynamics (DPD) for polymers, the force is usually computed as

$$\mathbf{F}_{ij} = \mathbf{F}_{ij}^{\mathrm{C}} + \mathbf{F}_{ij}^{\mathrm{D}} + \mathbf{F}_{ij}^{\mathrm{R}} + \mathbf{F}_{ij}^{\mathrm{spring}}. \tag{6}$$

With soft repulsive force

$$\mathbf{F}_{ij}^{\mathrm{C}} = \begin{cases} a_{ij}\left(1 - \frac{r_{ij}}{r_c}\right)\widehat{\mathbf{r}}_{ij} & r_{ij} < r_c \\ 0 & r_{ij} \geq r_c, \end{cases} \tag{7}$$

with repulsion parameter a_{ij}, cutoff radius r_c and unit vector $\widehat{\mathbf{r}}_{ij}$.

The dissipative force is given as

$$\mathbf{F}_{ij}^{\mathrm{D}} = -\gamma\left(1 - \frac{r_{ij}}{r_c}\right)^2 \left(\widehat{\mathbf{r}}_{ij} \cdot \mathbf{v}_{ij}\right)\widehat{\mathbf{r}}_{ij}, \tag{8}$$

with friction coefficient γ, the velocity difference between the particles, $\mathbf{v}_{ij}$, and θ being a random number between 0 and 1.

The random force is computed as

$$\mathbf{F}_{ij}^{\mathrm{R}} = \frac{\sigma\theta_{ij}}{\sqrt{\delta t}}\left(1 - \frac{r_{ij}}{r_c}\right)\widehat{\mathbf{r}}_{ij}, \tag{9}$$

with noise parameter σ.

The Frenkel spring force term is

$$\mathbf{F}_{ij}^{\mathrm{spring}} = -K_{\mathrm{s}}\left(r_{ij} - R_0\right)\hat{\mathbf{r}}_{ij}. \tag{10}$$

Following Smit and co-workers[12] we chose the spring constant $K_{\mathrm{s}} = 100 k_{\mathrm{B}} T$ and equilibrium distance R_0=0.80 DPD units, which was found in previous studies to be the first maximum of the pair correlation function of a pure monomer system.[13] All simulations were run using a number density set to 3 and an integration timestep of 0.025.

Espanõl and Warren[14] have shown that DPD will sample the canonical ensemble if

$$\gamma = \frac{\sigma^2}{2 k_{\mathrm{B}} T}. \tag{11}$$

Here we chose $\gamma = 4.5$.

Supplementary Table 1 | DPD cross interaction parameters. "S" denotes the solvent beads, "S2" denotes the beads belonging to the attractive layer of the surface, "S1" denotes the beads belonging to the repulsive core and "R", "Ta", "Tr", "W" represent the monomer beads.

bead *i*	bead *j*	a_{ij}
S	R	30
S	Ta	27.25
S	Tr	27.25
S	W	20
S	S1	25
S	S2	25
R	Ta	25
Ta	Tr	25
Tr	W	25
R	W	25
R	Tr	25
Ta	W	25
S2	R	15
S2	Ta	15
S2	Tr	20
S2	W	20
S1	R	75
S1	Ta	75
S1	Tr	75
S1	W	75
S2	S1	25

Surface model

The adsorption surface is modeled as face-centered cubic (fcc) lattice structure with an equilibrium bond length of 0.707 and a lattice cell length of $\sqrt[3]{4/3} \sim 1.1$ DPD length units. The surface model consists of an inner and outer layer region: The outer layers are one DPD length unit thick and constitute the attractive surface (S2), whereas the inner-layer (8 DPD length units) represents the repulsive core (S1). Note that the simulations are set up symmetrically and thus contain two outer layers for both sides of the surface. All fcc surfaces consisted of 10,240 DPD beads, corresponding to a total thickness of $\sim$10 DPD length units. The bond constant of the lattice was set to $100\,k_{\mathrm{B}}T$.

Supplementary Note 5 Molecular Simulations

Free energy of adsorption

Adsorption free energy simulations were initially prepared by solvating the fcc lattice surface model with 25,000 solvent beads (S) and the single chain polymer. This yielded a box dimension of approximately $17.6 \times 17.6 \times 37.8$ cubic DPD length units for each polymer system (3125 systems in total). Simulations were set up using Enhanced Monte Carlo (EMC) and run using the Large-scale Atomic/Molecular Massively Parallel Simulator (LAMMPS) code. Standard molecular dynamics (MD) simulations were performed for 1 M timesteps for all systems, prior to running steered MD and umbrella sampling simulations.

Both steered MD and subsequent umbrella sampling simulations were performed using the "ParticleSeparation" collective variable (CV) defined in the Software Suite for Advanced General Ensemble Simulations (SSAGES) code. The CV distance was defined as the distance between the fcc lattice center-of-mass and polymer center-of-mass along the z (normal) dimension. CV distances were set 1 DPD length units apart with minimum and maximum CV values of 1 to 12, respectively. This corresponded to 12 simulations being performed for each polymer system ($12 \cdot 3125$ polymers = 37,500 simulations in total). Steered MD simulations were first performed for 200,000 timesteps using a biasing spring constant of $15\,k_{\mathrm{B}}T$ to steer the polymer into the target CV windows. Umbrella sampling was then performed for 1 M timesteps using a spring constant of $15\,k_{\mathrm{B}}T$ for each umbrella. The weighted histogram analysis method (WHAM) was used to obtain the final potential of mean force (PMF) as a function of surface-polymer z center-of-mass separation distances. We found that setting the spring constant to $15\,k_{\mathrm{B}}T$ with CV separation distances of 1 DPD length unit provided good overlap between CV distance histograms used in WHAM. The adsorption free energy free energy of adsorption (ΔG_{ads}) was taken as the difference in free energy between free energy minimum along the z-dimension and the free energy of the polymer in the bulk phase, i.e., maximum z separation distance of 12. Note that we assume the Helmholtz free energy and Gibbs free energy as approximately equal (i.e., pV contributions are assumed negligible).

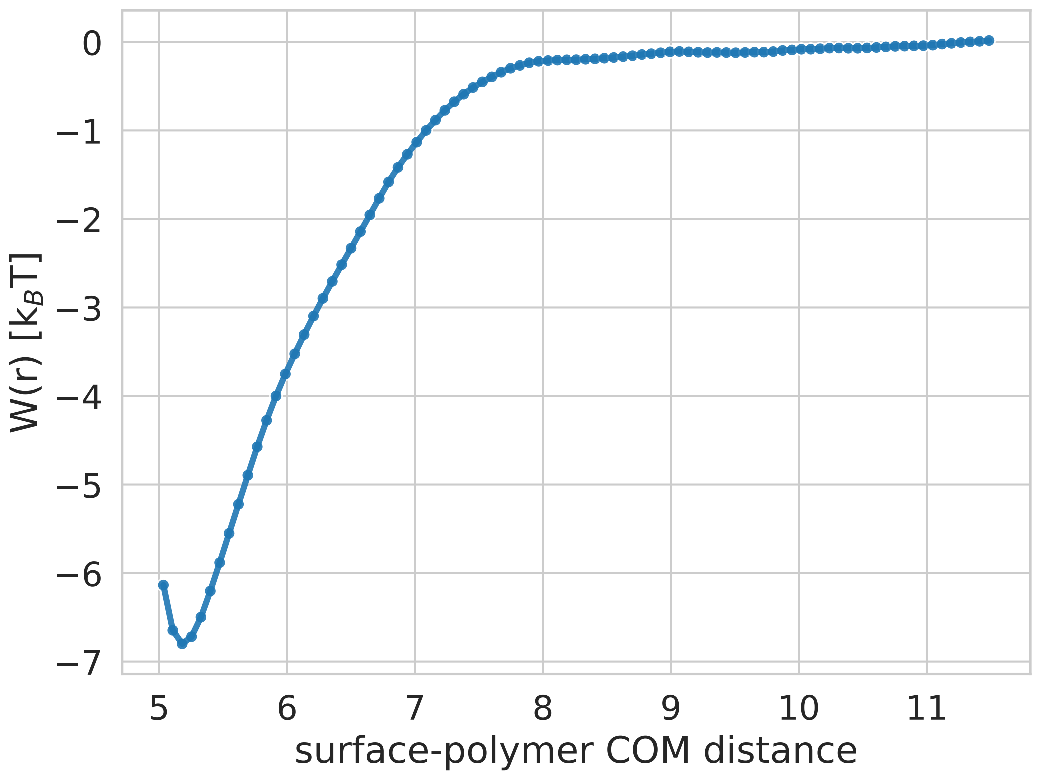

Supplementary Figure 6 | Potential of mean force (W) as a function of surface and polymer center-of-mass separation distance along the Z normal direction. ΔG_{ads} is taken as the difference in free energy between the bulk and minimum free energy.

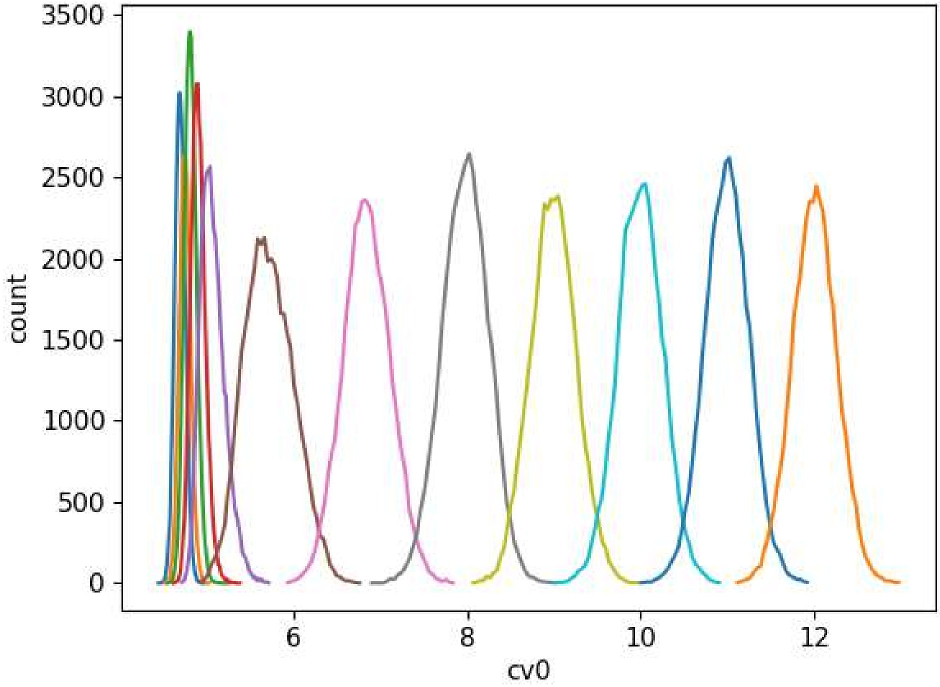

Supplementary Figure 7 | Umbrella sampling histograms obtained as a function of surface-polymer COM separation distance (cv0). Target CV separation distances were set to 1 DPD unit apart.

Dimer repulsion energy

Dimer repulsion energy simulations were prepared by solvating two identical polymers with 100,000 solvent beads, which corresponded to a box dimension of 32.2 × 32.2 × 32.2 cubic DPD length units. Steered MD and umbrella sampling simulations were performed using the "ParticleSeparation" CV in the x, y, and z dimensions, i.e., radial dimension. CV distances were set 1 DPD length units apart with minimum and maximum CV values of 0 to 11, respectively. This again corresponded to 12 simulations being performed for each polymer system (12 · 3125 polymers = 37,500 simulations in total). Similar to the adsorption free energy calculations, steered MD simulations and subsequent umbrella sampling simulations were performed for 200,000 and 1.5 M timesteps, respectively, with biasing spring constants of 15 $k_B T$. WHAM was used to obtain the final PMF curve as a function of polymer-polymer radial center-of-mass separation distance. Entropy corrections of 2 $\log(r)$ were also applied to the PMF curve. The dimer repulsion free energy repulsive free energy of polymer dimer (ΔG_{rep}) was taken as the

difference in free energy between the free energy at CV separation distances of 0 and the free energy at the maximum radial separation distances.

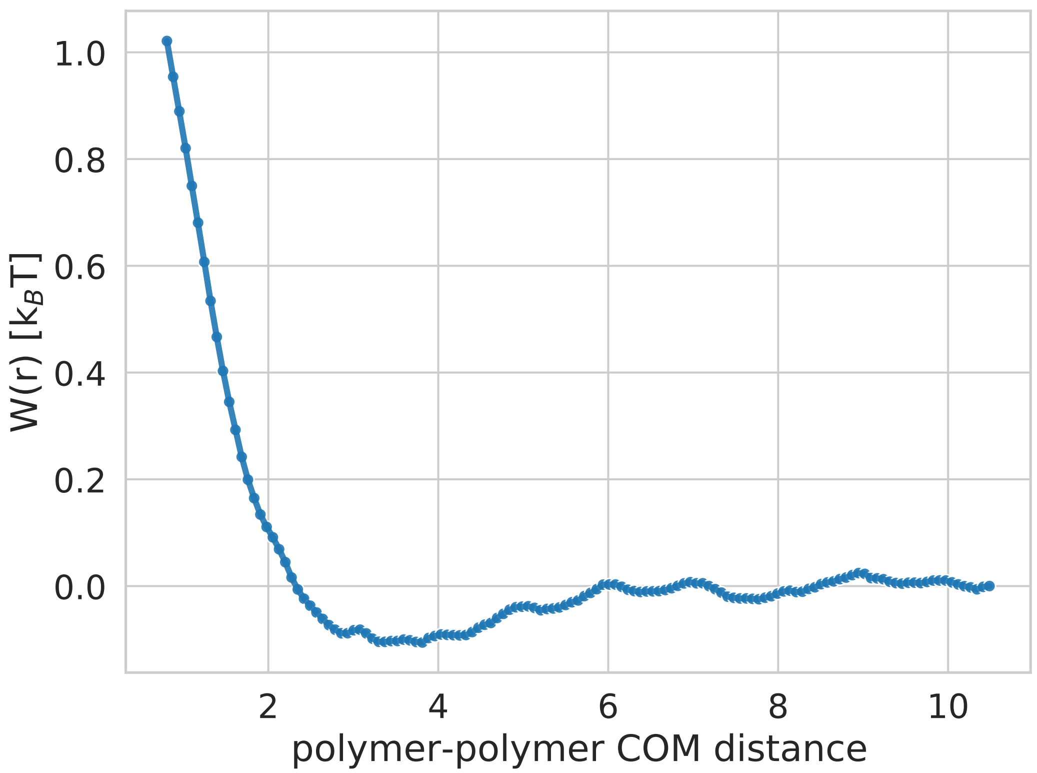

Supplementary Figure 8 | Potential of mean force (W) as a function of polymer and polymer center-of-mass separation distance along the radial (r) direction. ΔG_{rep} is taken as the difference in free energy between those at the maximum and minimum separation distances.

Radius of gyration

Radius of gyration simulations were prepared by solvating a single polymer with 5000 solvent beads corresponding to a box dimension of $11.9 \times 11.9 \times 11.9$ cubic DPD length unit. All simulations were run for 2 M DPD timesteps.

The radius of gyration (R_{g}) is computed as

$$R_{\text{g}}{}^2 = \frac{1}{M} \sum_i m_i (r_i - r_{\text{cm}})^2, \tag{12}$$

where M is the total mass, r_{cm} the center of mass and the sum is over all beads, using the `gyration` command in LAMMPS. R_{g} were output every 1000 steps and averaged over the entire simulation trajectory.

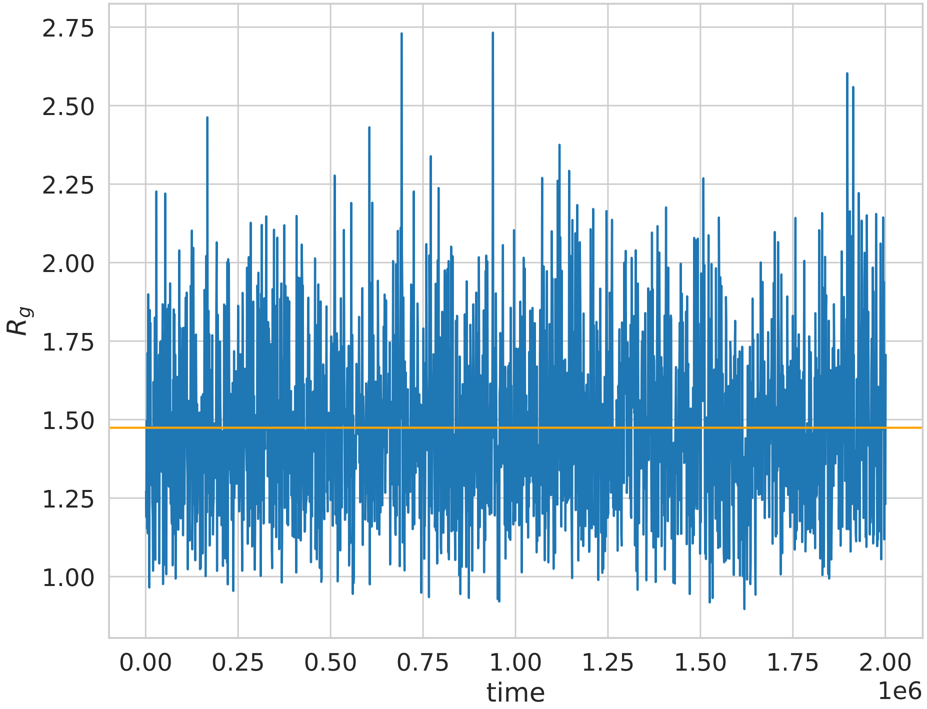

Supplementary Figure 9 | Radius of gyration R_g as a function of simulation timestep. $\langle R_g \rangle$ is taken as the average R_g over the entire simulation trajectory as indicated by the horizontal orange line.

Supplementary Note 6 Featurization

Polymer representation

Our text-based polymer notation uses the DPD bead types in place of atoms used in standard "SMILES". Parentheses can be used to reflect branching, however, we only consider linear polymers for the scope of this work.

Features considered in this work

For the linear polymers, we computed the following features from the monomer sequence. The feature names follow the ones we use in the dataset:

- `length`: degree of polymerization (number of beads)
- `head_tail_{bead}`: 1 if bead at head or tail of polymer, 2 if at head and tail, 0 otherwise
- `rel_shannon`: Shannon entropy of the polymer chain relative to the maximum possible entropy for a chain of the same length
- cluster statistics, describing clusters in which the same bead type is repeated:
  - `num_{bead}`: number of clusters for the bead type, relative total the total number of clusters
  - `total_clusters`: total number of clusters in a polymer chain
  - `max_{bead}`: maximum cluster size for the bead type
  - `min_{bead}`: minimum cluster size for the bead type
  - `mean_{bead}`: mean cluster size for the bead type
- `{bead}`: the frequency of a bead type in the polymer chain
- Statistics of the DPD interaction parameters:
  - `total_solvent`: the sum of the DPD interaction parameters of the polymer chain with the solvent
  - `total_surface`: the sum of the DPD interaction parameters of the polymer chain with the surface
  - `std_solvent`: the standard deviation of the DPD cross-interaction parameters of the polymer chain with the solvent
  - `std_surface`: the standard deviation of the DPD cross-interaction parameters of the polymer chain with the surface

Feature selection for the GPR surrogate models

For the final models we used the following features: num_[W], max_[W], num_[Tr], max_[Tr], num_[Ta], max_[Ta], num_[R], max_[R], [W], [Tr], [Ta], [R], rel_shannon, length. We used the same features for every surrogate model.

Supplementary Note 7 GPR surrogate model

We used the GPy[15] package to build and train the GPR and used the limited memory Broyden–Fletcher–Goldfarb–Shanno (L-BFGS) algorithm for hyper-parameter optimization.

For optimization of the hyperparameters we performed 20 random restarts with different initializations and used hyperparameters corresponding to the best maximum likelihood solution.

Utility of coregionalized models

Coregionalized models use multi-output kernels which have the following form:

$$\mathbf{B} \otimes \mathbf{K} = \begin{pmatrix} B_{1,1} \times \mathbf{K}(\mathbf{X}_1, \mathbf{X}_1) & \cdots & B_{1,D} \times \mathbf{K}(\mathbf{X}_1, \mathbf{X}_D) \\ \vdots & \ddots & \vdots \\ B_{D,1} \times \mathbf{K}(\mathbf{X}_D, \mathbf{X}_1) & \cdots & B_{D,D} \times \mathbf{K}(\mathbf{X}_D, \mathbf{X}_D) \end{pmatrix},$$

where $\mathbf{K}$ is a kernel function, $\mathbf{B}$ is regarded as the coregionalization matrix, and X_i represents the inputs corresponding to the i-th output. $\mathbf{B}$ allows the model to share information between outputs, which would not be possible for two independent kernels; it is hence especially valuable when one only has training data and sparse response data (i.e., missing data for some of the responses) for multiple objectives (see Supplementary Note 7). In case when all objectives are independent one would find $B_{ij} = 0 \, \forall \, i \neq j$. However, in the ϵ-PAL algorithm we are interested in learning any regularities that might exist in our design space as fast as possible.

Therefore we investigated how the coregionalized models perform compared to two separate GPR models given our training data. We chose R_g and ΔG_{ads} as targets. For this analysis, we employed the Matérn-3/2 kernel and intrinsic coregionalization model (ICM) for coregionalization. To remove the degeneracy in the variance hyperparameter (one in the ICM and another in the Matérn kernel), we constrained the variance of the Matérn kernel.

Two key-parameters for the surrogate models in the ϵ-PAL algorithm are the predicted variance (as this will influence how large our rectangles are) and the accuracy of the prediction (as ϵ-PAL will no utility if the model is not predictive). To investigate if a coregionalized kernel can be of use for our problem, we performed learning curve analysis, as our overall goal for active learning is to predict well with as little training data as possible. To

obtain error estimates, we performed the analysis 10 times with different random seeds. In Supplementary Fig. 10 we find that the coregionalized models outperform the two separate ones—notably for the smaller training set sizes, which we would be suitable for the ϵ-PAL algorithm.

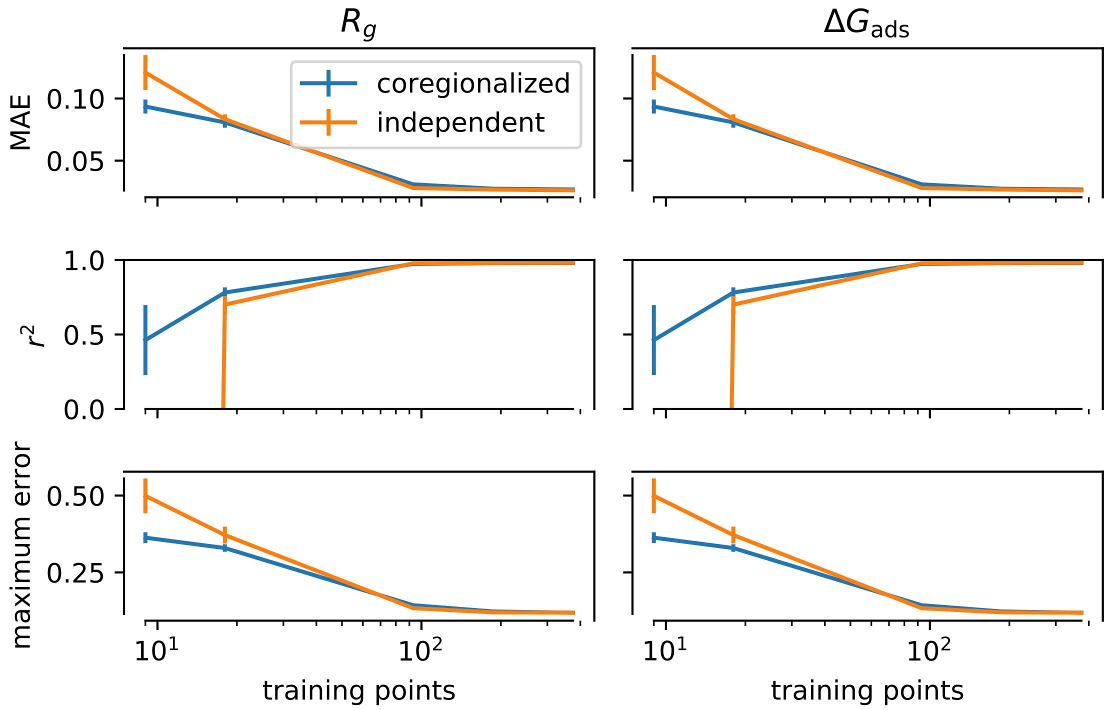

Supplementary Figure 10 | Learning curves for models with coregionalized kernel and independent models.

In Supplementary Fig. 11 we show the variance as a function of the training set size.

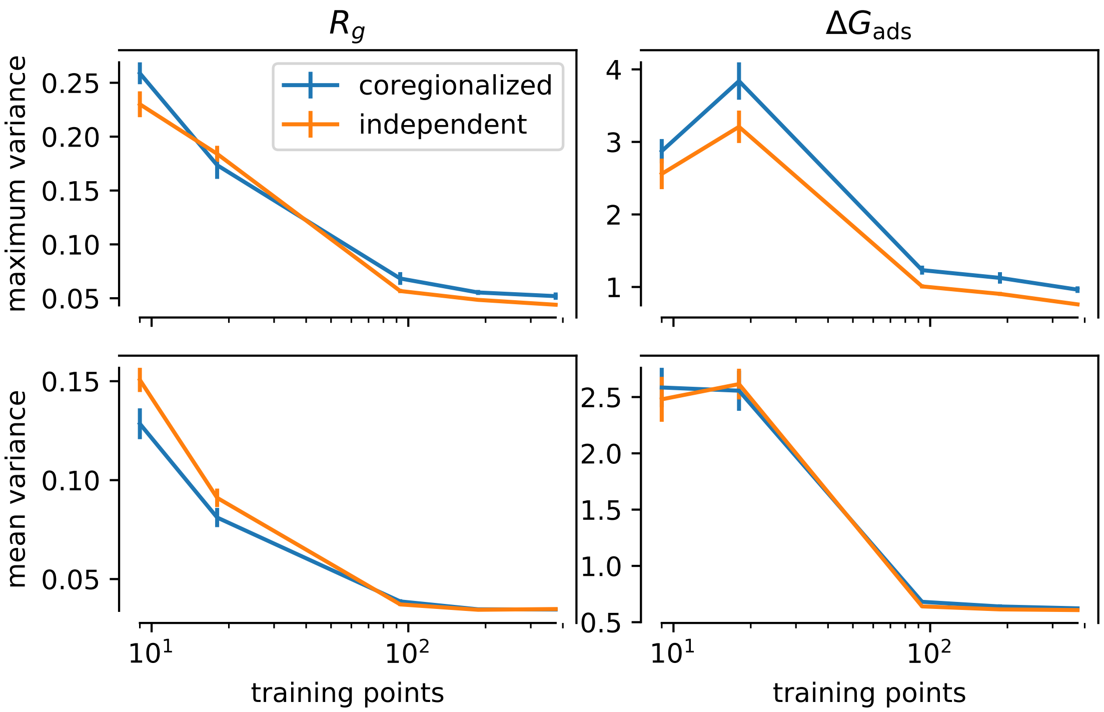

Supplementary Figure 11 | Predicted variance as a function of the training set size for models with coregionalized kernel and independent models.

In Supplementary Fig 12 we show the predictive performance of the GPR models trained with the ϵ-PAL active learning process.

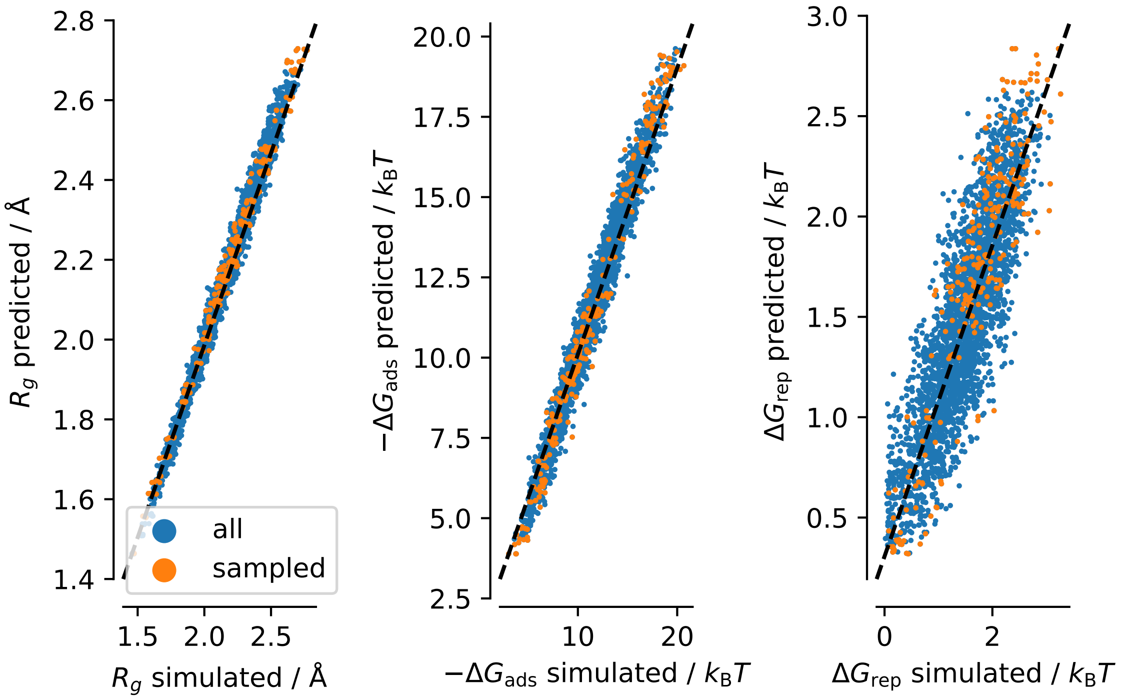

Supplementary Figure 12 | Predictive performance of the models trained with the ϵ-PAL active learning process.

In Supplementary Fig. 13 and 14 we show the SHAP summary plots for surrogate models trained with $\epsilon = 0.01$ and $\epsilon = 0.1$, respectively,

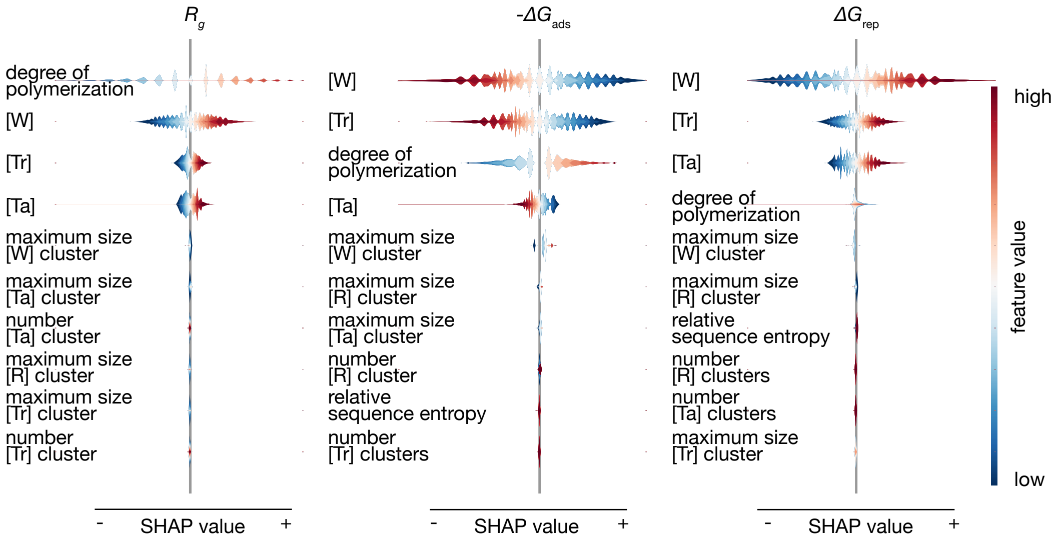

Supplementary Figure 13 | SHAP summary plot for a surrogate model (ICM, Matérn-5/2 kernel) trained over the course of a ϵ-PAL run with $\epsilon = 0.01$, $\delta = 0.05$ $\beta_{\text{scale}} = 0.05$.

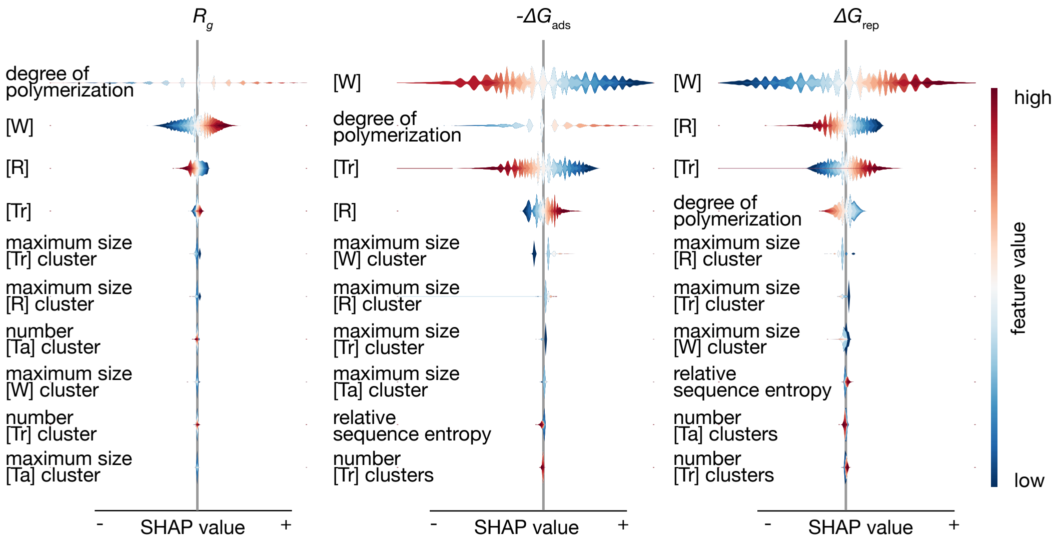

Supplementary Figure 14 | SHAP summary plot for a surrogate model (ICM, Matérn-5/2 kernel) trained over the course of a ϵ-PAL run with $\epsilon = 0.1$, $\delta = 0.05$ $\beta_{\text{scale}} = 0.05$.

Other model types

In Supplementary Figure 15 we compare the predictive performance of a rank 1 ICM model, rank 2 ICM model, independent GPR, the neural tangent kernel (NTK), and the neural network Gaussian process (NNGP). The NTK and NNGP were computed using the neural-tangents library.[16] The NTK and NNGP were based on the architecture [8, 8, 8] with errorfunction activation. We did not perform hyperparameter optimization for these neural network (NN)-based models.

For the GPR models we used a Matérn-5/2 kernel without ARD. We tested the models for predicting the R_g and ΔG_{ads}.

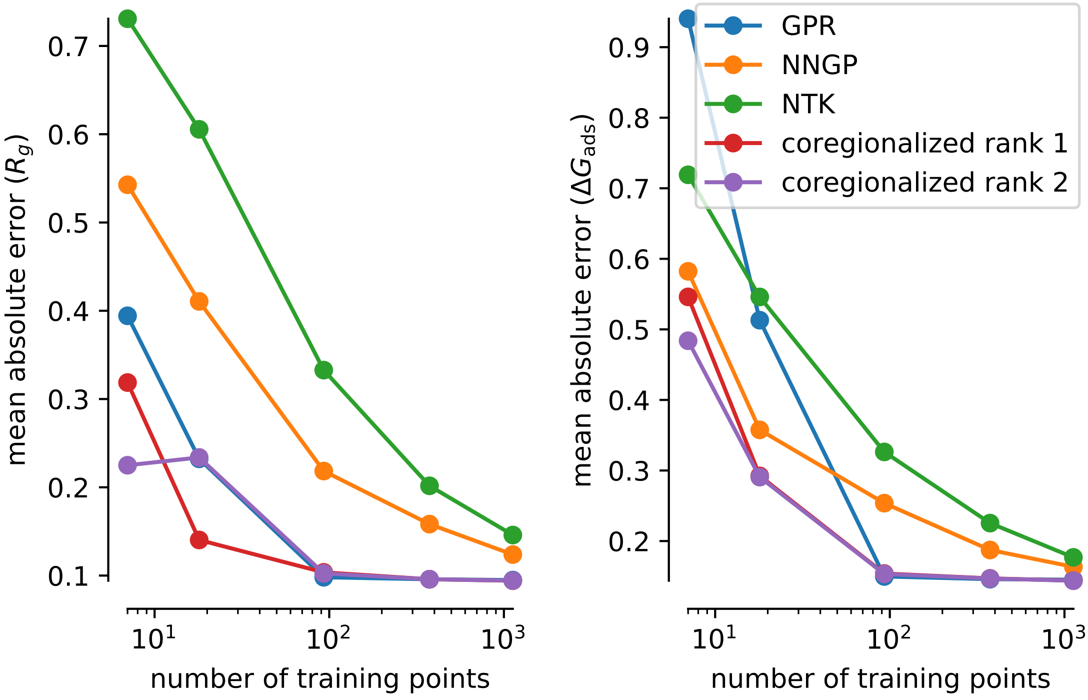

Supplementary Figure 15 | Learning curves for different model types. All GPR models were build using Matérn-5/2 kernels without ARD. The NNGP and NTK were build for a small, three-layer NN.

The learning curves indicate that in our case there is little difference between rank 1 and rank 2 coregionalized models.

Dealing with missing data

For the case study with missing data, we randomly discarded one-third of the simulation results for the dimer repulsion energy. The `PyePAL` code can with deal this situation with any kind of model but coregionalized models are particularly suitable as they can exploit correlations between the objectives and hence help with "filling in" the missing measurement. The progress of an active learning experiment in this setting is illustrated in Supplementary Figure 16.

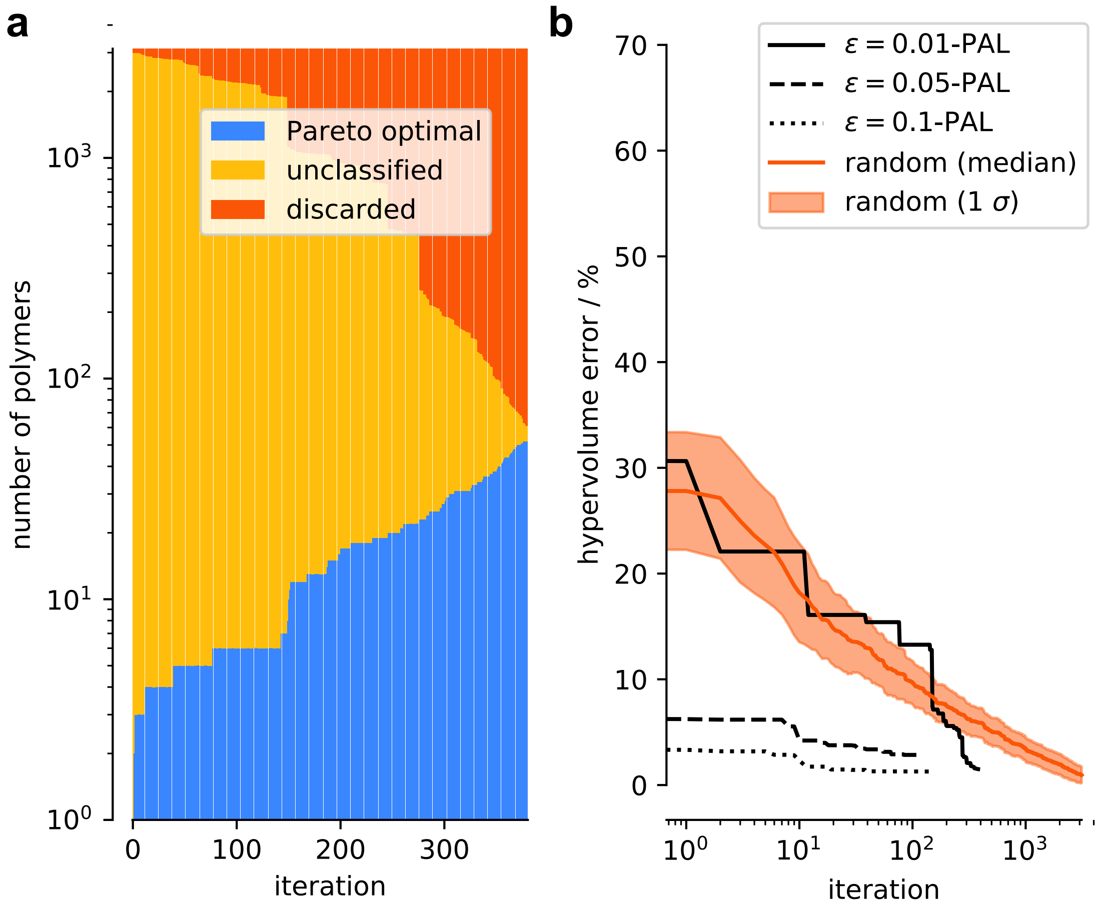

Supplementary Figure 16 | Classified points and hypervolume error as a function of the number of iterations. Using ICM with Matérn-5/2 kernel. Hypervolume error for random search (with all data present, i.e., no missing outputs) is shown for comparison. All search procedures were initialized using the same set of initial points, but vary substantially after only one iteration step. The hypervolume reference point for this figure is (-5, -5, -5).

Supplementary Note 8 Pareto optimal structures in feature and property space

Objective (property) space

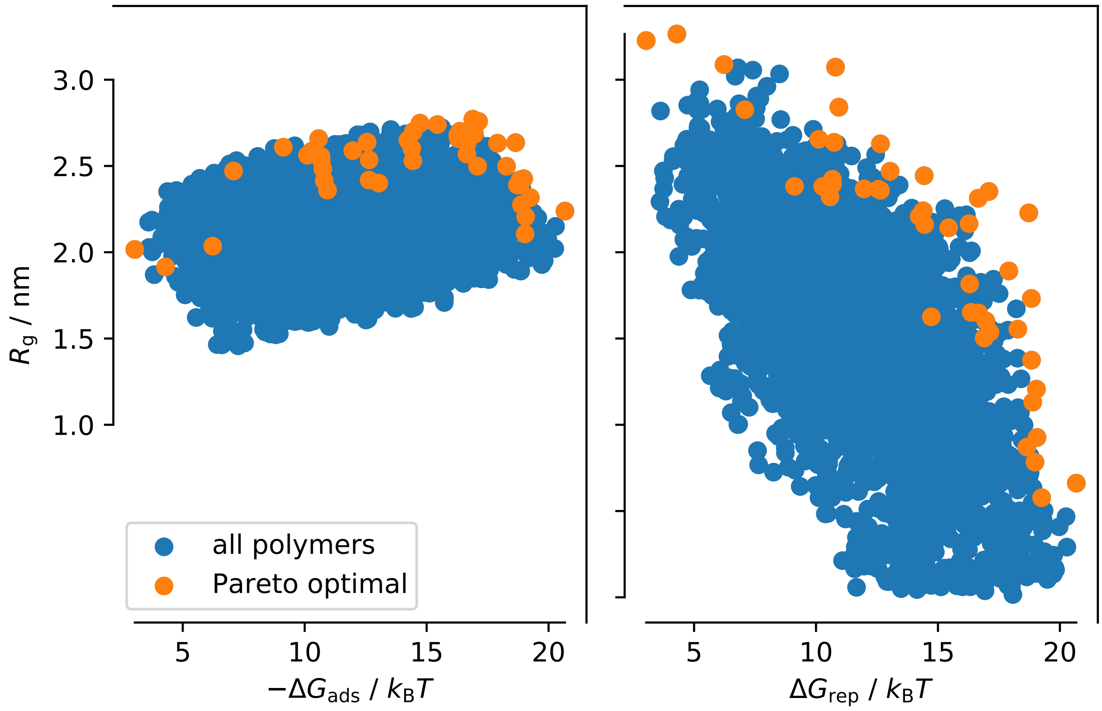

Supplementary Figure 17 | Overview of the design space and the Pareto optimal points in this design space.

Out of 3125 polymers in our experimental design, 73 are Pareto optimal according to our brute-force simulation results (Supplementary Fig. 17).

Feature space

To visualize feature space, we project the high-dimensional feature space onto two dimensions using principal component analysis (PCA) (Supplementary Fig. 18, using `scikit-learn`[17]) and uniform manifold approximation and projection for dimension reduction (UMAP) (Supplementary Fig. 19, using `umap-learn`[18]). We can observe that the Pareto optimal structures do not cluster in one region of feature space but are spread all over feature space.

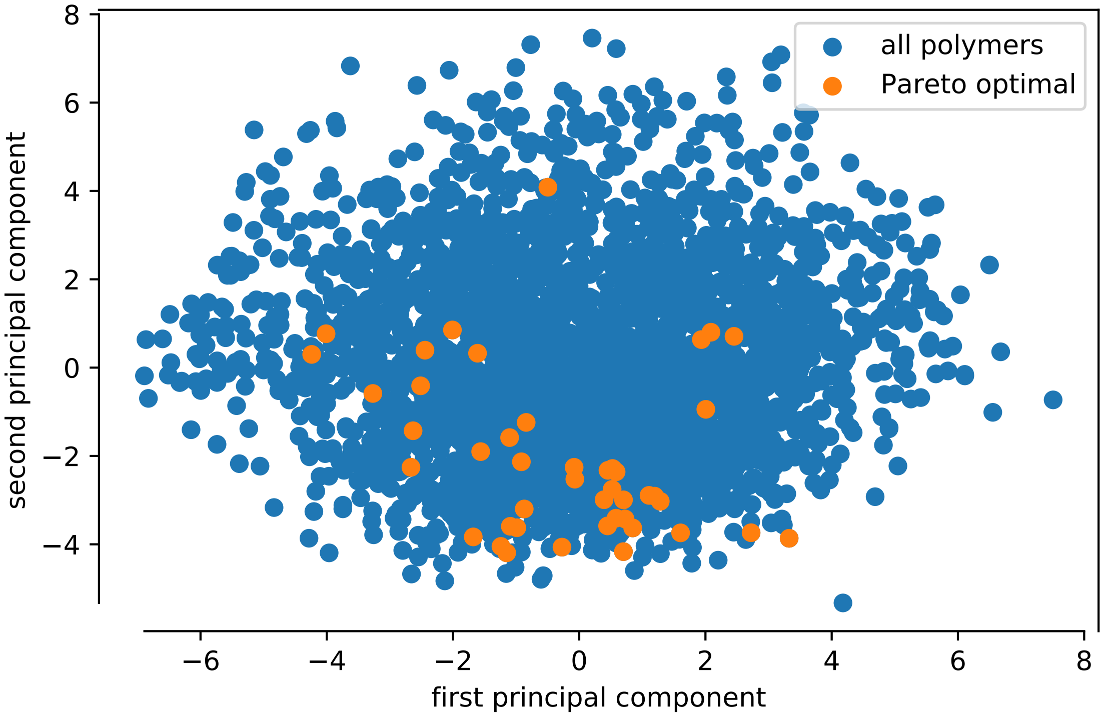

Supplementary Figure 18 | Projection of the feature space onto two dimensions using PCA.

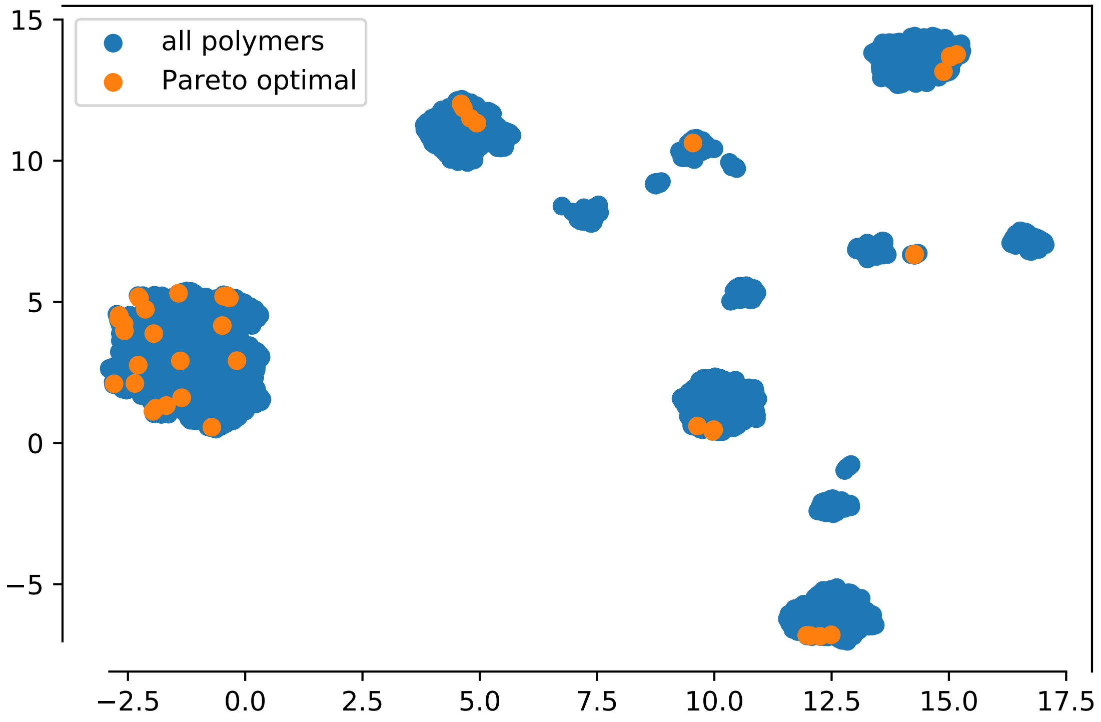

Supplementary Figure 19 | Projection of the feature space onto two dimensions using UMAP.

Supplementary Note 9 Hyperparameter tuning for the PAL algorithm

Influence of the initial training set

One crucial assumption for the theoretical bounds to be valid is that the true error is bounded by the estimate provided by the GPR (this can be problematic if the model is overconfident[19]). For this reason, we found empirically that it is practical to initialize the search with a diverse set of about that is large enough that the model is predictive. The minimum number of samples can be estimated using learning curve analysis. The influence of the number of initial points on the performance on our dataset is shown in Supplementary Figure 20.

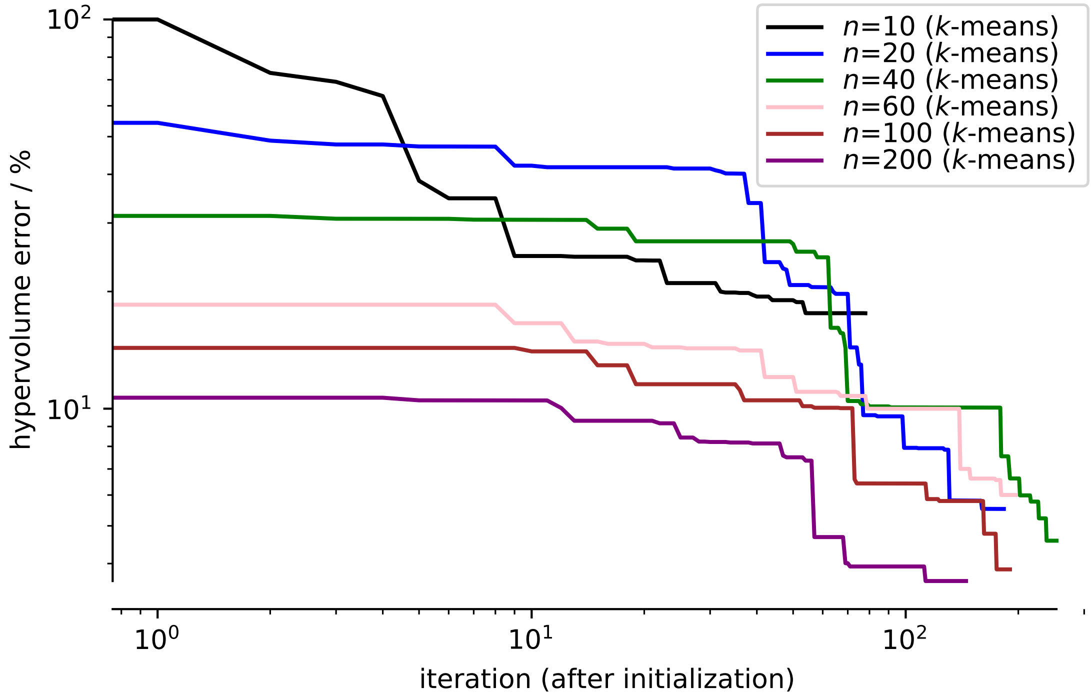

Supplementary Figure 20 | Influence of the number of initial points. Hypervolume error as a function of the number of initial points. We left $\epsilon = 0.05$, $\delta = 0.05$, $\beta_{\text{scale}} = 0.05$ fixed and sampled using k-means sampling. Note that a low number of initial samples (e.g., $n = 10$) can lead to unreliable results. This can be the case if the surrogate model is non-predictive and overconfident, causing misclassification of points early in the search. For this reason, the `PyePAL` package warns users when the cross-validation error is greater than the variance of the model. Moreover, the learning curves (Supplementary Fig. 15) indicate that the models have a large generalization error for $n \ll 60$. Hypervolumes were calculated using the nadir point as our reference point.

To ensure a good sampling of the initial space, we chose the n samples using a greedy MaxMin sampling, initialized with the point closest to the mean of the dataset.

Supplementary Figure 21 compares greedy MaxMin sampling with initialization based on k-means clustering. We find that the MaxMin sampling typically leads to faster convergence, but that the k-means sampling converges to lower errors.

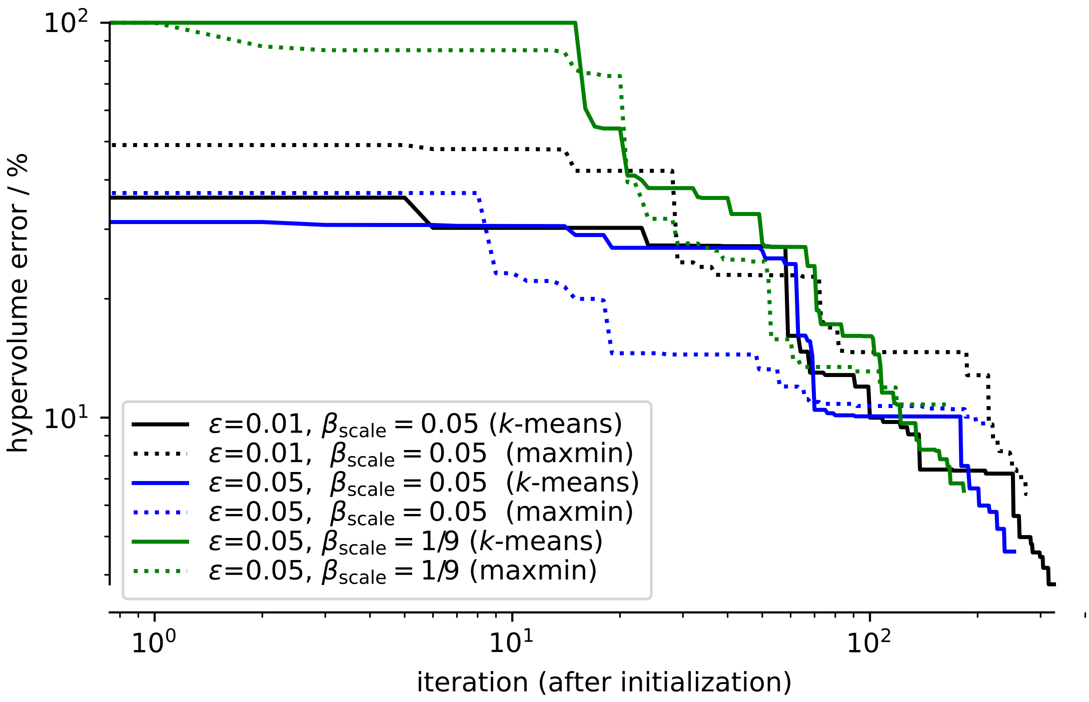

Supplementary Figure 21 | Influence of the sampling method that is used to create the initial set. Hypervolumes were calculated using the nadir point as our reference point.

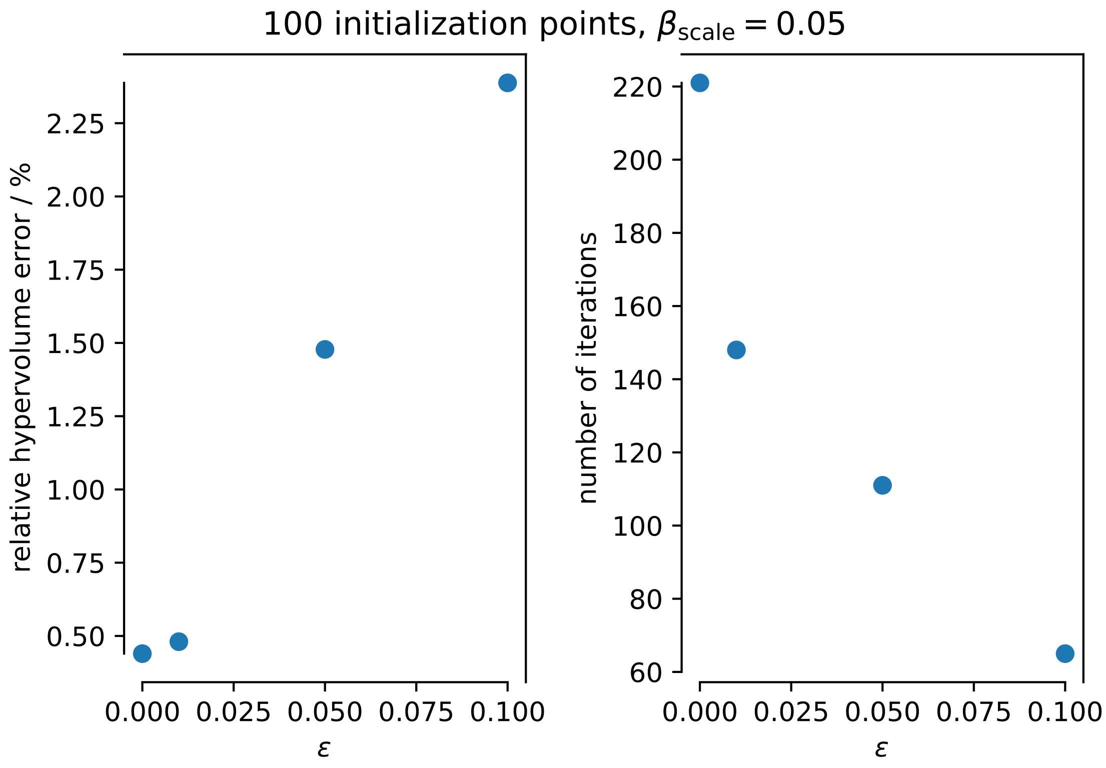

Supplementary Figure 22 | Relative hypervolume errors and total number of iterations of the ϵ-PAL algorithm as a function of ϵ ($\epsilon_i = \epsilon \, \forall i \in \{0, 1, 2\}$).

Supplementary Note 10 ε-PAL Implementation

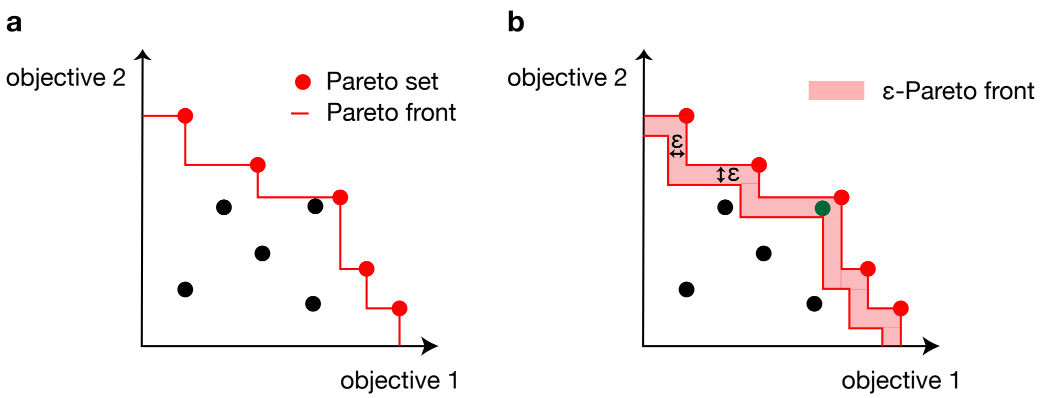

Supplementary Figure 23 | Pareto vs. ϵ-Pareto front for two objectives. a illustrates the concept of the Pareto set, i.e., the set of maximal points, and the Pareto, whereas **b** shows a ϵ-Pareto front. ϵ Pareto optimality would also be given if the Pareto set includes the green point instead of the neighboring red one.

Overview of the algorithm

The input of the ϵ-PAL algorithm is the initial design space E, the priors for the GPR models, as well as the hyperparameters ϵ_i and δ. Additionally, we use a scaling parameter (β_{scale}) of the scaling parameter for the hyperrectangle (β_t) as the theoretical value tends to be too conservative. For most of our ϵ-PAL runs, we set the hyperparameters δ=0.05, ϵ_i, and scaled β_t by $1/20$. In our `PyePAL` package, we allow the user to chose custom schedules for the optimization of the hyperparameters of the GPR, a batch size, and exclude high-variance points from the classification step.

For the subsequent discussion we need to define the following symbols, which mostly follow the notation from Zuluaga et al.:

- design space (E): finite set of point from which we sample
- (ϵ-accurate) Pareto set (P): the solution we aim to find
- set of unclassified points (U): in the first iteration $U_0 = E$
- set of discarded points (D): points for which we can say with high confidence that they are not ϵ Pareto optimal
- using the standard deviation and mean vectors predicted by the GPR we use the β_t to compute a conservative uncertainty hyperrectangle of point $\mathbf{x}$ ($Q_{\mu,\sigma,\beta}(\mathbf{x})$)

- iterative intersection of the hyperrectangles gives us uncertainty region of point $\mathbf{x}$ ($R_t(\mathbf{x})$): $R_t(\mathbf{x}) = R_{t-1} \cap Q_{\mu,\sigma,\beta}(\mathbf{x})$

It is also useful to compare the concept of Pareto optimality with the one of ϵ-accurate Pareto dominance, assuming a maximization problem.

- Pareto dominance: We say that y dominates y' iff $y \succeq y'$, i.e., y is no worse than y' in all objectives and strictly better in at least one objective
- ϵ-Pareto dominance: We relax the definition to $y + \epsilon \succeq y'$, which we also write as $y \succeq_\epsilon y'$

Note that in contrast to the original implementation we do not require knowledge of the value ranges of the objectives to compute the uncertainty hyperrectangles. In general, this is not known *a priori*. That is, instead of computing the tolerance as $\epsilon_j r_j$, where r_j is the range of objective j, we use $\epsilon_j \mu_j$ where μ_j is the prediction for objective j. Additionally, this ensures that the tolerance is proportional to the value of the μ_j (and is not inflated/deflated depending on the range). We compare both behaviors in Supplementary Fig. 24. We find the adaptive tolerances converge to lower errors and to also show lower errors in the initial and intermediate iterations. This is particularly pronounced if we do not set the uncertainties of the sampled points to zero but instead use the uncertainties predicted by the GPR.

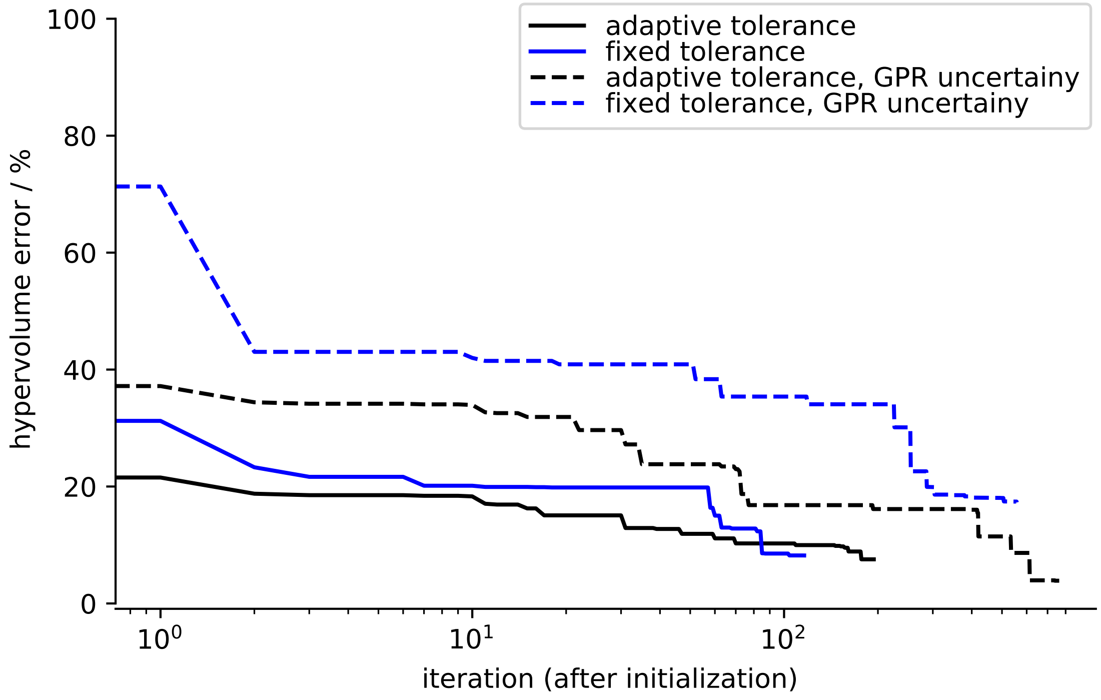

Supplementary Figure 24 | Fixed tolerances vs. adaptive tolerances. To expedite this experiment we used a $\lg_2$ spaced schedule for hyperparameter optimization (in contrast to the linearly space schedule used in the rest of this work). We left $\delta = 0.05$ and $\beta_{\text{scale}} = 0.05$ fixed, used a Matérn-5/2 kernel without ARD, and initialized with 40 points sampled using greedy farthest point sampling. Hypervolumes were calculated using the nadir point as our reference point.

For calculation of the hypervolumes we use code from the `nevergrad` library.[20] Note that hypervolumes are not used in the algorithm itself but logged to monitor our algorithm convergence.

Moreover, we implemented the prediction error $\varepsilon(\hat{P}, P)$[21]

$$\varepsilon(\hat{P}, P) = \frac{1}{\|P\|} \sum_{i}^{\|P\|} \min_{x' \in \hat{P}} \max_{1 \leq j \leq p} \frac{\left(f_j(\mathbf{x}) - f_j(\mathbf{x}')\right) \cdot 100}{r_j}, \tag{13}$$

where $\hat{P}$ is the predicted Pareto front, p the number of objectives, r_j is the range of the values for objective j, and $\|P\|$ the number of points in the set of Pareto optimal points, P.

For more detail on the ϵ-PAL algorithm, we refer the reader to the original implementation.[21;22]

Initialization To initialize the GPR a few samples from the design space need to be first evaluated. In practice, this can be done by selecting k samples

closest to the centroids of a k-means clusters or by using a greedy MaxMin sampling approach (which we initialize with the median or mean).

Modelling As discussed in section 7, we use Gaussian processes as surrogate models and the mean and variance of the posterior function to construct hyperrectangles. We decided to add the frequency of hyperparameter optimization of the GPR models as a hyperparameter for the ϵ-PAL algorithm.

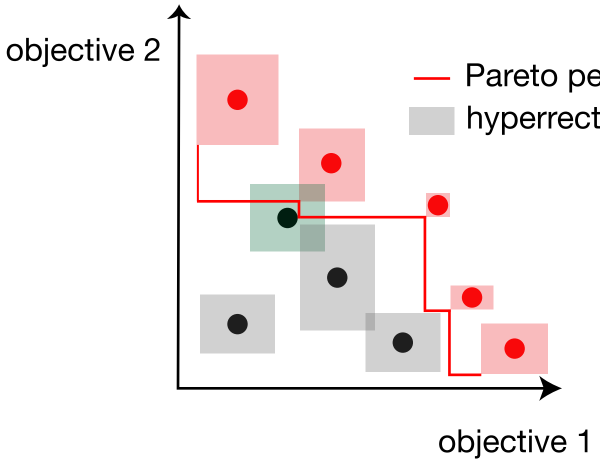

Supplementary Figure 25 | Illustration of the set of Pareto pessimistic points. In the discarding step, we would keep the point with the green uncertainty region $R_t(\mathbf{x})$ as we cannot say with certainty that it is lower than the Pareto pessimistic p_{pess} front but will discard all points with gray hyperrectangles. In the discarding step, we build two different Pareto pessimistic fronts. First, only from the points we already classified as ϵ-Pareto optimal. Then, followed by ones where we consider the union of ϵ-Pareto optimal and unclassified points.

Discarding Any point is removed from the unclassified set if its optimistic outcome is ϵ dominated by the pessimistic outcome of another point. More specifically, we first consider the Pareto pessimistic set $p_{\text{pess}}(P)$ and then the Pareto pessimistic set $p_{\text{pess}}(P \cup U)$, where a Pareto pessimistic set is defined as the set of points $\mathbf{x}$ for which there is no other point $\mathbf{x}'$ such that $\min\left(R_t(\mathbf{x})\right) \preceq \min\left(R_t(\mathbf{x}')\right)$. For this reason, we are guaranteed that the discarding step is safe since there always will be a point that ϵ dominates the discarded points. This is a key feature that is of particular importance for materials design and discovery.

Identification of ϵ Pareto optimal points A point $\mathbf{x}$ belongs with high probability to the output set of ϵ-accurate Pareto points if there is no other point $x' \in P \cup U$ such that $\max\left(R_t(\mathbf{x}')\right) \preceq_\epsilon \min\left(R_t(\mathbf{x})\right)$.

Sampling In the sampling stage the next sample is one of the Pareto optimal or unclassified points with the highest uncertainty w_t:

$$w_t(\mathbf{x}) = \max_{\mathbf{y},\mathbf{y}' \in R_t(\mathbf{x})} \left\| \frac{y - y'}{\hat{y}} \right\|_2 . \tag{14}$$

Note that the algorithm does not sample from the discarded points. To ensure scale invariance, we rescale the uncertainty in each direction by the mean prediction, i.e., we use the coefficient of variation for sampling. Beyond de-biasing the search, this change can have an impact on the performance of the algorithm as shown in Supplementary Figure 26.

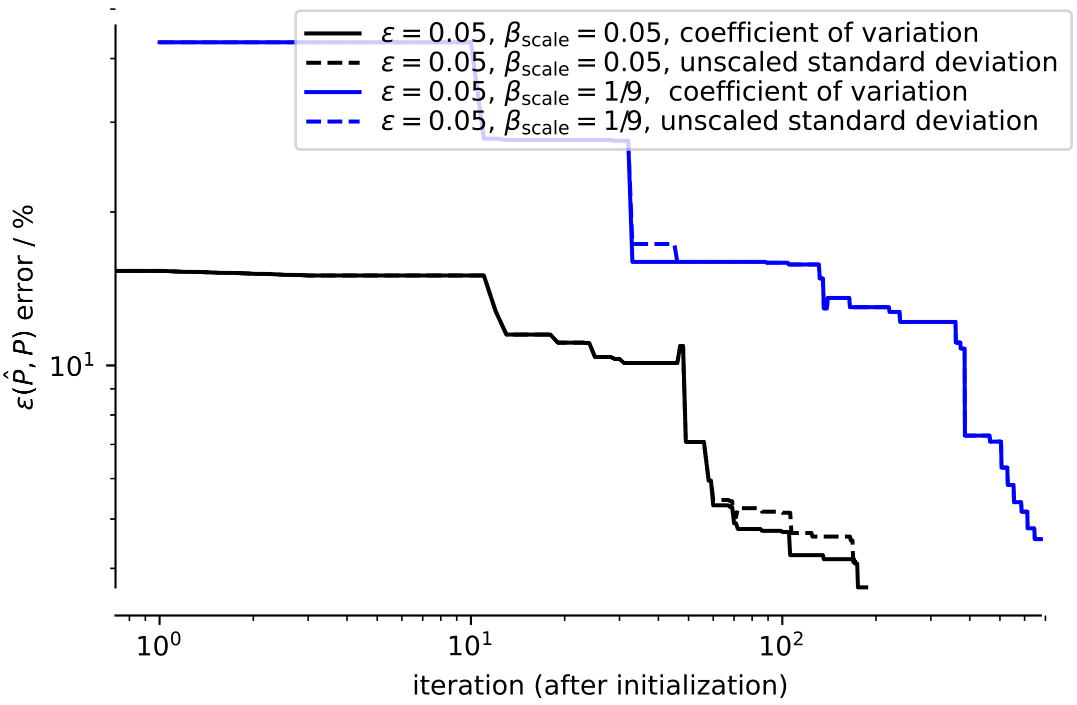

Supplementary Figure 26 | Influence of scaling the variance on the performance of the algorithm. Using the coefficient of variation instead of the unscaled uncertainty marginally improves the performance in our test cases.

As the choice of the aggregation function (with which the different objectives are combined into one scalar for the sample step) is not unique, we compared the performance of the Frobenius norm (default in the `PyePAL`

package, used for this work) with the mean and median (Supplementary Figure 27).

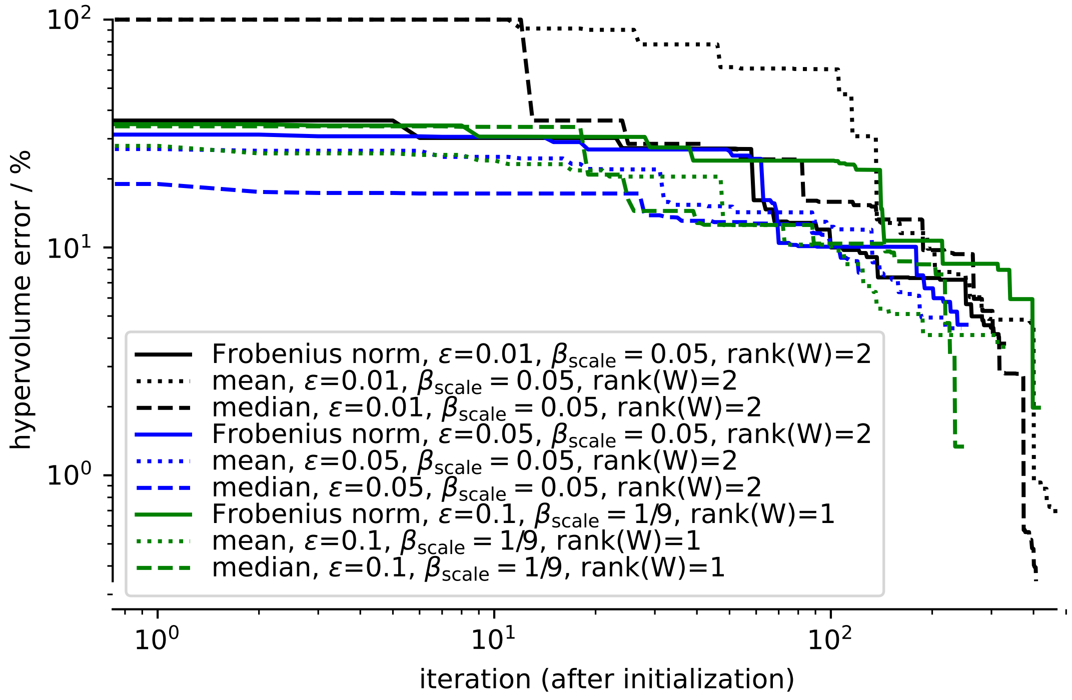

Supplementary Figure 27 | Influence of the aggregation function. We observe that the Frobenius norm leads to faster termination of the search, e.g., compared to median aggregation, which leads to lower final hypervolume errors. Hypervolumes were calculated using the nadir point as our reference point.

In our implementation we, by default, will use the measured mean and standard deviations instead of the predictions of the surrogate model. From Supplementary Fig. 28 we see that replacing the GPR uncertainty for the sampled points with zero greatly expedites the convergence.

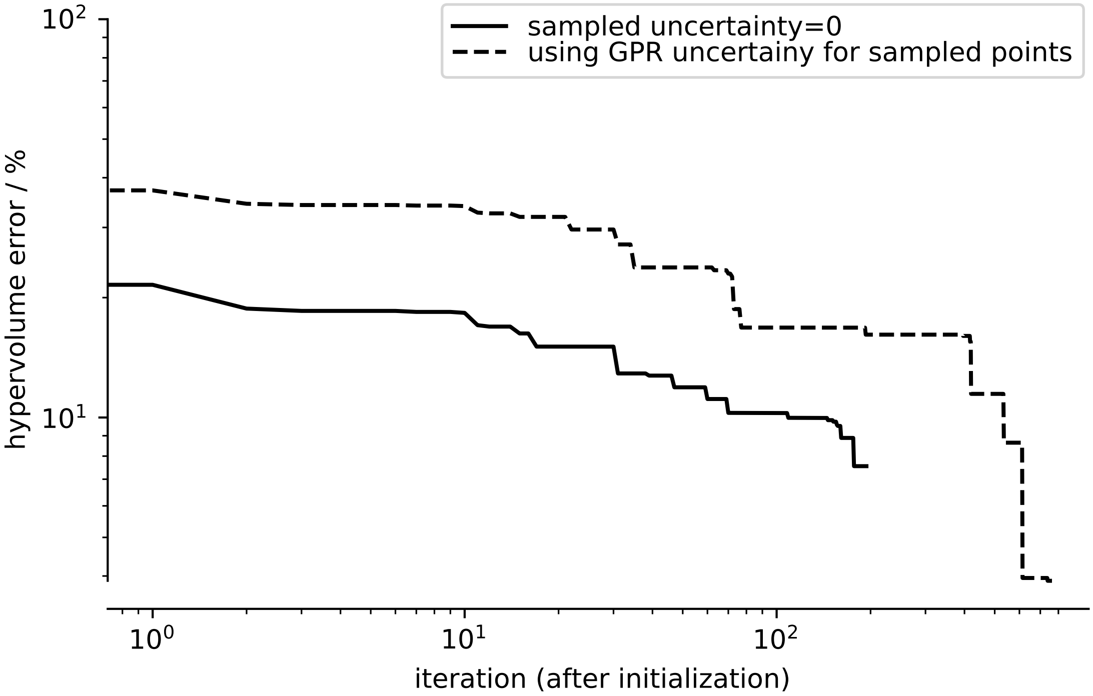

Supplementary Figure 28 | Replacing the uncertainty with the measured uncertainty (here asummed to be zero). To expedite this experiment we used a $\lg_2$ spaced schedule for hyperparameter optimization (in contrast to the linearly space schedule used in the rest of this work). We left $\delta = 0.05$, $\epsilon = 0.05$, and $\beta_{\text{scale}} = 0.05$ fixed, used a Matérn-5/2 kernel without ARD, and initialized with 40 points sampled using greedy farthest point sampling.

We chose to not implement sampling methods that require retraining of the models for all potential candidates (e.g., expected error reduction[23;24]) as those techniques would extremely increase the computational cost of the algorithm (retraining and evaluating the model(s) for every possible new sample, averaged over all possible labels), even though those techniques might mitigate the tendency of uncertainty sampling[25] to sample outliers.

Stopping The algorithm stops when all points are either discarded or classified as Pareto optimal, i.e., if $U = \emptyset$.

Batch sampling

Batch sampling can be beneficial if simulations or experiments can be parallelized and when a sequential scheme is too time-consuming. The original ϵ-PAL scheme samples only one sample per iteration. In this work, we did not employ batch sampling. However, in our `PyePAL` package, we allow the user to perform ϵ-PAL in batch mode. We use a greedy approximation and

sample the n next best samples according to the selection criterion rather than just sampling one point. Note that the greedy approximation lowers the efficiency of the exploration of the space.

Multiple ε

For some applications it is often preferred to have the different tolerances for ϵ on the Pareto front for each objective. To account for such flexibility, we use one ϵ_i per dimension i.

Theoretical guarantees

Zuluaga et al.[21] showed that the ϵ-PAL algorithm comes with some guarantees for the quality of the solution. For that reason, one assumes that the functions which are modeled with the GPR are arbitrary functions from the reproducing kernel Hilbert space (RKHS) with some associated kernel k. Additionally, the noise of the samples is assumed to have zero mean conditioned on the history and to be bounded by σ. For appropriate choice of hyperparameter β_t, Zuluaga et al. proved that an ϵ-accurate Pareto front can be found in a bounded number of iterations with probability $1 - \delta$, where δ is also a hyperparameter that can be specified by the user.

Limitations

It is well known that kernel methods tend to need stringent feature selection.[26] In case one only has access to a high-dimensional feature space with noisy data, the GPR models might have a too low predictive performance for a decent convergence of ϵ-PAL. Future work could investigate Monte-Carlo dropout based surrogate models.[27]
Furthermore, ϵ-PAL, operates on a finite design space, i.e., it will not find a design that is not in the set of possible designs it is provided with as input. Therefore, any continuous design space needs to be discretized.
Additionally, since we require the returned points to be ϵ-Pareto optimal, this approach can require many iterations until the first point is classified as ϵ-Pareto optimal if the predicted variance stays high even after many iterations.

Sensitivity of the hypervolume error to the choice of the reference point

Since the reference point for the hypervolume calculation is a user defined parameter we explored a range of different settings. For the reference points we considered the minimum of our design space (sampled using the DoE), some intermediate point $(-5, -5, -5)$ and a point orders of magnitude larger than the objectives $(-1000, -1000, -1000)$. In Supplementary Fig. 29 we compared the convergence behavior to the median of 100 random explorations of the design space.

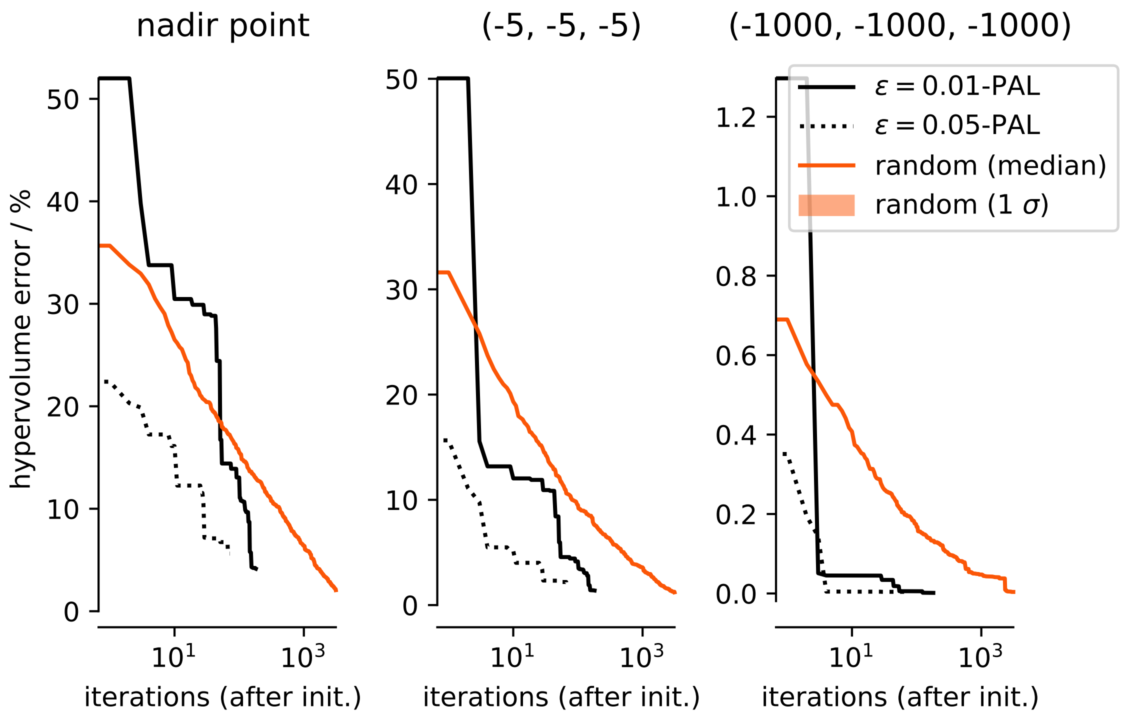

Supplementary Figure 29 | Convergence behavior compared to random search for different hypervolume reference points. The curves are calculated for the same runs as shown in Fig. 6 in the main text.

Supplementary Note 11 Inverse design

We use the term "inverse design" to refer to finding a valid polymer that maximizes the outputs of our models.[28]

Surrogate model

To be able to perform practical inverse design, we cannot use all the features used in this work. For example, the Shannon entropy feature, provided a length and set of characters, is partially invertible but would require a complex constraint for our optimization algorithm. This would make the search inefficient.
To avoid this issue, we trained gradient boosted decision tree (GBDT) models with reduced feature sets to predict the predictions of the GPR models. For those surrogate models, we optimized the hyperparameters with Bayesian optimization. The details can be found under sweep ids f2cteo9b, 704xptpt, 1vwsrp8b on the wandb platform). Note that the use of surrogate models that are trained on the predictions on other models is a commonly used technique to interpret models.[29;30]

Genetic algorithm

We also attempted to use particle swarm optimization (PSO) as implemented in the `pyswarm`[31] package but found better results with genetic algorithm (GA), for which we used the `geneticalgorithm` Python package.[32] The main advantage of GA over PSO for our optimization problem is that GA allows for a more natural treatment of mixed datatype (integer and real) optimization.

Since not all possible features correspond to valid polymers and we are mostly interested in novel polymers, we used the following fitness function

$$\mathcal{L}(\mathbf{x}) = -10\,\frac{\hat{y}(\mathbf{x})}{\overline{y}_{\text{train}}} + \mathrm{CP}(\mathbf{x}) + \mathrm{NIVP}(\mathbf{x}) + \alpha\mathrm{NP}(\mathbf{x}), \tag{15}$$

where $\hat{y}$ is the prediction of the model. CP and NIVP are penalty terms that penalize structures with unphysical cluster size features and those of which are not invertible, respectively. The penalties have the following form

$$\mathrm{CP}(\mathbf{x}) = \begin{cases} \texttt{max\_bead\_type} > \texttt{length} \cdot \texttt{bead} & +30 \\ \text{else} & 0 \end{cases} \forall\,\texttt{bead}, \tag{16}$$

where we used the feature notation from section 6, and

$$\mathrm{NIVP}(\mathbf{x}) = \begin{cases} \text{at least one valid monomer seq. generated} & 0 \\ \text{else} & 50, \end{cases} . \tag{17}$$

where we use the algorithm described in section 11 to iteratively attempt the mapping from features to a bead sequence.

The last term in eq. 15 is a penalty term that increases the loss for structures that are similar to the ones from our database to encourage the GA to explore new areas of chemical space:

$$\mathrm{NP}(\mathbf{x}) = \max\left(\overline{y}_{\text{train}}\left(\overline{\min \|\mathbf{x}_{\text{train,i}} - \mathbf{x}_{\text{train}}\|} - \min \|\mathbf{x} - \mathbf{x}_{\text{train}}\|\right), -\overline{y}_{\text{train}}\right). \tag{18}$$

To consider a wide balance between exploration and exploitation we swept through $\alpha = \{0, 0.1, 0.5, 1, 2, 10, 20, 50, 100\}$ with multiple random restarts for each α.

We considered the feature bounds listed in Supplementary Table 2.

Supplementary Table 2 | Feature bounds for the GA.

feature	lower bound	upper bound	type
`length`	16	48	`int`
`max_{bead}`	0	36	`int`
`{bead}`	0	1	`real`

For the elitist GA we used the hyperparameters listed in Supplementary Table 3.

Supplementary Table 3 | Parameters used for the GA.

parameter	value
restarts	3
maximum number of iterations	300
elite ratio	varied {0, 0.01, 0.05, 0.07}
population size	300
mutation probability	0.1
crossover probability	0.8
parents portion	0.1
crossover type	uniform
early stopping after	500 iterations without improvement

Mapping back to valid monomer sequences

For our promising solutions, we enumerated possible monomer sequences. To map back to physically valid bead sequences we round the bead numbers and degree of polymerization found in GA to integers, and calculate the maximum size of the clusters to capture the topological features of the polymer (pool of beads and clusters).

To efficiently evaluate if any permutation of the given characters can build a valid monomer sequence, we use a backtracing algorithm.[33] This algorithm sequentially evaluates if the addition of a character from the pool can still provide a valid monomer sequence, based on the constraints on the number of beads and the maximum size of the clusters. If the addition is successful, we pop this character from the pool and continue recursively calling the function until the pool of candidate beads is empty.

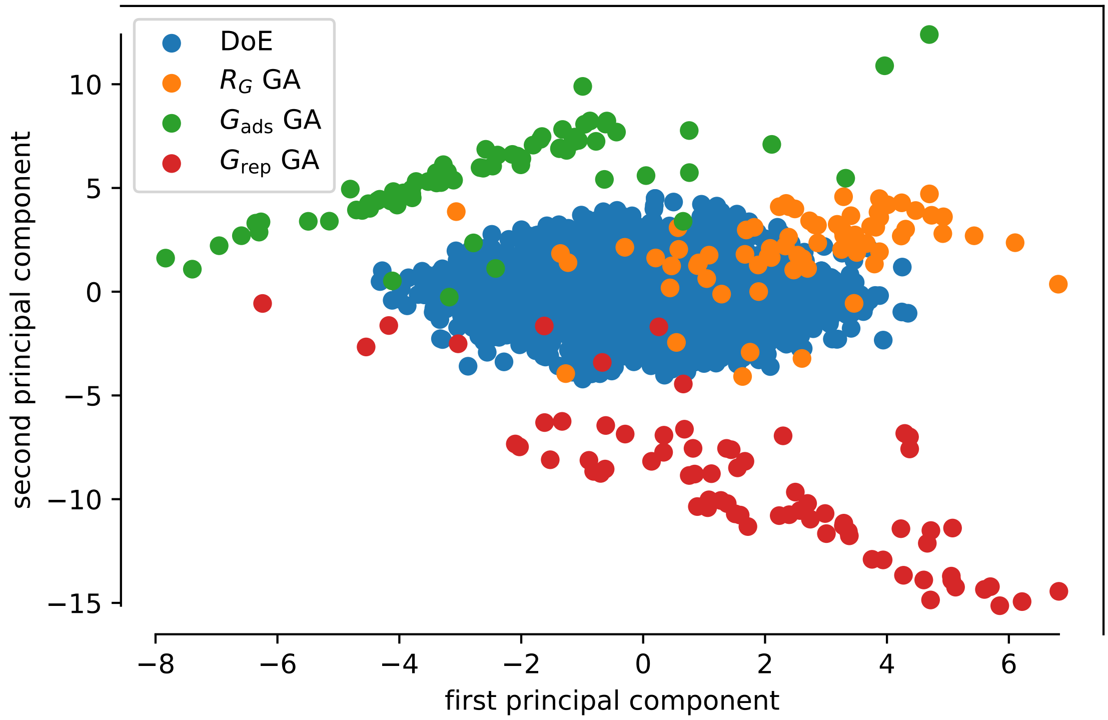

Supplementary Figure 30 | Projection of the generated feature sets on the first two principal components of the database.

Since none of the predictions we made for the polymers we found in the "inverse design" step dominated a point from the Pareto front we found in the subspace sampled with DoE, we did not perform any additional simulations.

In Supplementary Table 4 we compare hypervolumes found with the GA, and ϵ-PAL on the subspace sampled with DoE.

Supplementary Table 4 | Hypervolume of design space sampled with DoE and Pareto front found using the GA.

reference point	hypervolume original DoE	hypervolume GA	PyePAL (ϵ=0.01)
minimum (1.46, 3.04, 1.61×10^{-3})	268	166	257
(-5,-5,-5)	1536	1283	1515
(-1000, -1000, -1000)	1 026 829 209	1 025 214 020	1 026 813 775

Acronyms

D set of discarded points. 33

E design space. 33

P (ϵ-accurate) Pareto set. 33, 36, 37

$Q_{\mu,\sigma,\beta}(\mathbf{x})$ uncertainty hyperrectangle of point $\mathbf{x}$. 33, 34

$R_{\mathbf{g}}$ radius of gyration. 17, 21, 25

$R_t(\mathbf{x})$ uncertainty region of point $\mathbf{x}$. 34, 36, 37

U set of unclassified points. 33, 36, 37, 39

$\Delta G_{\mathbf{ads}}$ free energy of adsorption. 14, 15, 21, 25

$\Delta G_{\mathbf{rep}}$ repulsive free energy of polymer dimer. 16, 17

β_t scaling parameter for the hyperrectangle. 33, 40

ARD automatic relevance determination. 8, 25, 26, 35, 39

CV collective variable. 14, 16, 17

DoE design of experiments. 9, 10, 41, 44, 45

DPD dissipative particle dynamics. 12–14, 16, 17, 19

EGO efficient global optimization. 7

EI expected improvement. 3, 4, 7, 8

fcc face-centered cubic. 13, 14

GA genetic algorithm. 42–45

GBDT gradient boosted decision tree. 42

GPR Gaussian process regression. 8, 21, 23, 25, 26, 30, 33–36, 38, 40, 42

ICM intrinsic coregionalization model. 21, 24, 25, 27

L-BFGS limited memory Broyden–Fletcher–Goldfarb–Shanno. 21

LAMMPS Large-scale Atomic/Molecular Massively Parallel Simulator. 14, 17

MD molecular dynamics. 14, 16

NN neural network. 25, 26

NNGP neural network Gaussian process. 25, 26

NTK neural tangent kernel. 25, 26

PAL Pareto active learning. 8, 21–25, 32, 33, 35, 36, 39, 40, 44

PCA principal component analysis. 28, 29

PMF potential of mean force. 14, 16

PSO particle swarm optimization. 42

RKHS reproducing kernel Hilbert space. 40

UMAP uniform manifold approximation and projection for dimension reduction. 28, 29

WHAM weighted histogram analysis method. 14, 16

Supplementary References

1. Zitzler, E.; Brockhoff, D.; Thiele, L. The Hypervolume Indicator Revisited: On the Design of Pareto-Compliant Indicators Via Weighted Integration. Evolutionary Multi-Criterion Optimization. Berlin, Heidelberg, 2007; pp 862–876.

2. Wagner, T.; Emmerich, M.; Deutz, A.; Ponweiser, W. On Expected-Improvement Criteria for Model-Based Multi-Objective Optimization. Parallel Problem Solving from Nature, PPSN XI. Berlin, Heidelberg, 2010; pp 718–727.

3. del Rosario, Z.; Rupp, M.; Kim, Y.; Antono, E.; Ling, J. Assessing the Frontier: Active Learning, Model Accuracy, and Multi-Objective Candidate Discovery and Optimization. *J. Chem. Phys.* **2020**, *153*, 024112.

4. Moffaert, K. V.; Nowé, A. Multi-Objective Reinforcement Learning Using Sets of Pareto Dominating Policies. *J. Mach. Learn. Res.* **2014**, *15*, 3663–3692.

5. Forrester, A. I. J.; Sóbester, A.; Keane, A. J. *Engineering Design via Surrogate Modelling: A Practical Guide*, 1st ed.; Wiley, 2008.

6. Keane, A. J. Statistical Improvement Criteria for Use in Multiobjective Design Optimization. *AIAA Journal* **2006**, *44*, 879–891.

7. Janet, J. P.; Ramesh, S.; Duan, C.; Kulik, H. J. Accurate Multiobjective Design in a Space of Millions of Transition Metal Complexes with Neural-Network-Driven Efficient Global Optimization. *ACS Cent. Sci.* **2020**, *6*, 513–524.

8. Ishibuchi, H.; Imada, R.; Setoguchi, Y.; Nojima, Y. How to Specify a Reference Point in Hypervolume Calculation for Fair Performance Comparison. *Evol Comput* **2018**, *26*, 411–440.

9. Frazier, P. I. A Tutorial on Bayesian Optimization. *arXiv:1807.02811 [cs, math, stat]* **2018**,

10. Frazier, P. I. In *Recent Advances in Optimization and Modeling of Contemporary Problems*; Gel, E., Ntaimo, L., Shier, D., Greenberg, H. J., Eds.; INFORMS, 2018; pp 255–278.

11. Wu, J.; Frazier, P. In *Advances in Neural Information Processing Systems 32*; Wallach, H., Larochelle, H., Beygelzimer, A., d\textquotesingle Alché-Buc, F., Fox, E., Garnett, R., Eds.; Curran Associates, Inc., 2019; pp 9813–9823.

12. Rekvig, L.; Kranenburg, M.; Vreede, J.; Hafskjold, B.; Smit, B. Investigation of Surfactant Efficiency Using Dissipative Particle Dynamics. *Langmuir* **2003**, *19*, 8195–8205.

13. Goicochea, A. G. Adsorption and Disjoining Pressure Isotherms of Confined Polymers Using Dissipative Particle Dynamics. *Langmuir* **2007**, *23*, 11656–11663.

14. Español, P.; Warren, P. Statistical Mechanics of Dissipative Particle Dynamics. *Europhys. Lett.* **1995**, *30*, 191–196.

15. GPy, GPy: A Gaussian process framework in python. http://github.com/SheffieldML/GPy, 2020.

16. Novak, R.; Xiao, L.; Hron, J.; Lee, J.; Alemi, A. A.; Sohl-Dickstein, J.; Schoenholz, S. S. Neural Tangents: Fast and Easy Infinite Neural Networks in Python. International Conference on Learning Representations. 2020.

17. Pedregosa, F. et al. Scikit-Learn: Machine Learning in Python. *J. Mach. Learn. Res.* **2011**, *12*, 2825–2830.

18. McInnes, L.; Healy, J.; Melville, J. UMAP: Uniform Manifold Approximation and Projection for Dimension Reduction. *arXiv:1802.03426 [cs, stat]* **2018**,

19. Karvonen, T.; Wynne, G.; Tronarp, F.; Oates, C. J.; Särkkä, S. Maximum Likelihood Estimation and Uncertainty Quantification for Gaussian Process Approximation of Deterministic Functions. *arXiv:2001.10965 [cs, math, stat]* **2020**,

20. Rapin, J.; Teytaud, O. Nevergrad - A gradient-free optimization platform. 2018; https://GitHub.com/FacebookResearch/Nevergrad.

21. Zuluaga, M.; Krause, A.; Püschel, M. ε-PAL: An Active Learning Approach to the Multi-Objective Optimization Problem. *J. Mach. Learn. Res.* **2016**, *17*, 1–32.

22. Zuluaga, M.; Sergent, G.; Krause, A.; Püschel, M. Active Learning for Multi-Objective Optimization. Proceedings of the 30th International Conference on Machine Learning. Atlanta, Georgia, USA, 2013; pp 462–470.

23. Roy, N.; McCallum, A. Toward Optimal Active Learning through Sampling Estimation of Error Reduction. Proceedings of the Eighteenth

International Conference on Machine Learning. San Francisco, CA, USA, 2001; p 441–448.

24. Cohn, D. A.; Ghahramani, Z.; Jordan, M. I. Active Learning with Statistical Models. Proceedings of the 7th International Conference on Neural Information Processing Systems. Cambridge, MA, USA, 1994; p 705–712.

25. Lewis, D. D.; Gale, W. A. *SIGIR '94*; Springer London, 1994; pp 3–12.

26. Jablonka, K. M.; Ongari, D.; Moosavi, S. M.; Smit, B. Big-Data Science in Porous Materials: Materials Genomics and Machine Learning. *Chem. Rev.* **2020**, *120*, 8066–8129.

27. Gal, Y.; Ghahramani, Z. Dropout as a Bayesian Approximation: Representing Model Uncertainty in Deep Learning. *arXiv:1506.02142 [cs, stat]* **2016**,

28. Sanchez-Lengeling, B.; Aspuru-Guzik, A. Inverse Molecular Design Using Machine Learning: Generative Models for Matter Engineering. *Science* **2018**, *361*, 360–365.

29. Ribeiro, M. T.; Singh, S.; Guestrin, C. "Why Should I Trust You?": Explaining the Predictions of Any Classifier. *arXiv:1602.04938 [cs, stat]* **2016**,

30. Molnar, C. *Interpretable Machine Learning - A Guide for Making Black Box Models Explainable*; 2019; https://christophm.github.io/interpretable-ml-book/.

31. Lee, A. Pyswarm. 2020; https://github.com/tisimst/pyswarm.

32. Solgi, R. M. Geneticalgorithm. 2020; https://github.com/rmsolgi/geneticalgorithm.

33. Knuth, D. *The art of computer programming*; Addison-Wesley: Reading (Mass.) Menlo Park (Calif.) London etc, 1968.
